# Enhanced Ion-Exchange Properties of a Complex Microporous Uranyl Borophosphate

**DOI:** 10.1021/acs.inorgchem.5c04481

**Published:** 2025-12-09

**Authors:** Yucheng Hao, Xu Jingli, Thomas E. Albrecht, Shuao Wang, Rüdiger-A. Eichel, Evgeny V. Alekseev

**Affiliations:** † School of Energy Materials and Chemical Engineering, Hefei University, Hefei 230000, China; ‡ Department of Chemistry and Nuclear Science and Engineering Center, Colorado School of Mines, Golden, Colorado 80401, United States; § School for Radiological and Interdisciplinary Sciences (RAD-X) and Collaborative Innovation Center of Radiation Medicine of Jiangsu Higher Education Institutions, Suzhou, Jiangsu 215123, China; ∥ Institute of Energy Technologies (IET-1), Forschungszentrum Jülich GmbH, 52428 Jülich, Germany

## Abstract

An unusual microporous uranyl borophosphate has been prepared under mild hydrothermal conditions, namely, Cs_3_(UO_2_)_3_[B­(PO_4_)_4_]­(H_2_O)_0.5_ denoted as CUPB1. It crystallizes in the chiral space group *P*4_1_2_1_2. The three-dimensional (3D) open framework of CUPB1 is constructed by [B­(PO_4_)_4_]^9–^ fundamental building blocks (FBBs) linked by UO_7_ pentagonal bipyramids into nanoscale size (∼12.2 Å × 12.2 Å × 11.7 Å) uranyl borophosphate cages of U_12_P_24_B_8_. The Cs^+^ cations are disordered and are located near the cage windows. Free void volume in CUPB1 is ∼59% based on the single-crystal X-ray diffraction data, which makes it a highly porous compound. Moreover, the porosity and flexibility of the Cs^+^ cations in the framework make it exchangeable with most monovalent and divalent cations in aqueous solutions. We have studied the ion-exchange properties in detail with the environmentally relevant cations Pb^2+^, Co^2+^, and Ni^2+^, as well as the key nuclear fission products Sr^2+^ and Ba^2+^, at both room temperature and ∼70 °C. The simple synthetic route, microporous structure, thermal stability, vibrational spectroscopy, and ion-exchange properties are discussed in detail.

## Graphical abstract

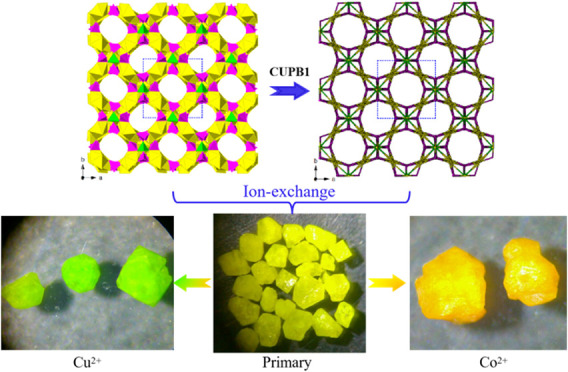

## 1 Introduction

Open framework materials are of high interest field due to their fascinating structures and important applications.^1^ They can be used in areas as diverse as ion exchangers, gas adsorption and storage, catalysts, nonlinear optical materials, etc.^2^ zeolites^3a^ and metal–organic framework^3b^ (MOF) materials are typical examples, which always possess highly porous framework structures. Zeolites are characterized as the oxo-tetrahedral framework structures, such as aluminosilicates,^4a^ aluminogermanates,^4b^ aluminophosphates,^4c^ and zinc phosphates.^4^ ^d^ MOFs are defined as compounds consisting of metal ions or clusters coordinated to organic ligands to form three-dimensional (3D) framework structures.^3b^ However, the thermal stabilities and expenses of MOFs have limited the vast applications in the industry.^5,6^

The substitution of boron for aluminum/silicon in zeolites has long been a topic of interest, because the resulting oxo-borates have complex structural chemistry and remarkable properties.^7,8^ Borates possess the intrinsic structural complexity, owing to the ability of the B^3+^ ion to occur in triangular BO_3_ and tetrahedral BO_4_ coordination environments in the same structure.^9^ These two basic units via corner or edge sharing polymerized into clusters, chains, scaffolds, and even 3D open framework structures.^10,11^ Boralite, Zn_4_O­(BO_2_)_6_,^12^ is a rare example of complete boron substitution for both Si and Al, which is a direct topological analogue of the aluminosilicate sodalite-type framework found in Na_4_OH­(AlSiO_4_)_3_.^13^ The zeolite-like network of Na_2_Co_2_B_12_O_2_ ^14^ is the first infinite borate containing a discernible tunnel structure, in which the Na^+^ within the tunnels are mobile, and exchangeable with Li^+^ preservation of the original crystal morphology. The actinide borate, [ThB_5_O_6_(OH)_6_]­[BO­(OH)_2_]·2.5H_2_O (NDTB-1),^15a^ which has a porous super tetrahedral 3D framework structure, possess exceptional ion-exchange properties toward TcO_4_^–^.^15b,16^ The incorporation of those mixed oxo-anions, such as [SiO_4_]^4–^, [AlO_4_]^5–^, [GeO_4_]^4–^, [PO_4_]^3–^, etc., gave rise to new families of borates derivative materials.^17,18^ From the previously reported results,^17^ the introduction of mixed oxo-anions into borates system, the resulting structures are favorite to form higher dimensional 3D open frameworks. For example, borosilicate Cs_2_B_4_SiO_9_ ^19^ and its basic B_4_O_10_ groups are connected with neighboring SiO_4_ tetrahedra forming a 3D network, which is a deep-ultraviolet nonlinear optical crystal. The organic templated aluminoborate, [CH_3_NH_3_]_1.5_[CH_3_CH_2_CH_2_NH_3_]_0.5_[H_2_O]_5_[Al­(B_5_O_10_)],^20^ shows an unprecedented 3D intersecting channel system. The framework is built from the strict alternation of B_5_O_10_ clusters and AlO_4_ tetrahedra. Borogermanate, SrGe_2_B_2_O_8_,^21^ has a 3D open anionic framework structure, composed of B_2_O_7_ and Ge_2_O_7_ dimers with alternate connections. The large B–Ge 8-ring tunnels that hold the Sr^2+^ cations make the Sr^2+^ cations movable at higher temperatures, which has a certain ion-exchange property with the toxic Cd^2+^ cations in the solution.^22^

Borophosphates (BPOs) have been widely studied for their diverse structural architectures and broad applications.^23^ Since the first zeolite-like borophosphate (C_2_H_10_N_2_)­[CoB_3_P_3_O_12_(OH)_12_]_18_ was reported in 1996, a plethora of open framework borophosphates have been prepared under different conditions.^23^ The borophosphate frameworks are quite complex and feature various anionic partial structures, such as oligomeric units, 1D chains and ribbons, 2D sheets, and 3D open frameworks.^23^ These abundant BPO frameworks were formed from the different templated cations, which are alkali, alkaline earth, transition metal, lanthanide elements, as well as the organic templates.^24,25^ Among them, only a few materials own the ion-exchange properties, for example, open framework vanadium borophosphate Na_2_[VB_3_P_2_O_12_(OH)]·2.92H_2_O,^26a^ chromium/aluminum borophosphate Na_8_[Cr_4_B_12_P_8_O_45_(OH)_4_]­[P_2_O_7_]·8H_2_O,^26b^ and aluminum borophosphate Na_3_[Al_2_B_6_P_4_O_22_(OH)_3_]­(H_2_O)_6_.^27^ More importantly, the open frameworks of borophosphates are capable of maintaining 5f-elements (actinides) of different oxidation states, as well as providing a stable matrix for interrogating isotopes with short half-lives.^28^ To the best of our knowledge, there are quite limited open framework actinide borate phosphates/borophosphates^29^ have been reported compared to the actinide-free series. Borate phosphates, such as U_2_(BO_4_)­(PO_4_) and Th_2_(BO_4_)­(PO_4_), along with borophosphates like Ag_2_(NH_4_)_3_[(UO_2_)_2_{B_3_O­(PO_4_)_4_(PO_4_H)_2_}]­H_2_O, K_5_(UO_2_)_2_[B_2_P_3_O_12_(OH)]_2_(OH)­(H_2_O)_2_, Pu­(H_2_O)_3_(B_2_(OH)­(H_2_O)­(PO_4_)_3_), etc., exhibit remarkable diversity in their structural chemistry.^29,30^ The synthesis of novel microporous open framework actinide borate phosphates/borophosphates is a great challenge. Compared to transition metal or lanthanide MOFs, the uranyl cation (UO_2_^2+^) has a series of unique properties: high stability, fixed linear geometry, which directs specific structural motifs, and its relevance to the chemistry of nuclear materials. Unlike many MOFs,^5,6^ which can be unstable in water or radiation fields, dense inorganic uranyl compounds reported, for example, by zur Loye group, like A_2_(UO_2_)­B_2_O_5_(A = Cs, Rb, K),^31b^ A_4_[(UO_2_)_3_(PO_4_)_2_O_2_]­(A = alkali metals),^31c^ and Cs_2_(UO_2_)­Al_2_O_5_,^31d,31d^ are often highly resistant to radiation damage and can be stable in aqueous environments, making them more suitable for applications like radioactive waste immobilization or separation.

Herein, the first cesium uranyl borophosphate Cs_3_(UO_2_)_3_[B­(PO_4_)_4_]­(H_2_O)_0.5_(CUPB1) was prepared and reported. It is a microporous material and features a novel 3D open framework structure. An unprecedented spherical uranyl borophosphate cage, U_12_P_24_B_8_, can be observed within its network. The unique structural architecture has been analyzed, together with thermogravimetric and differential scanning calorimetry characterizations, Raman spectroscopy, and exceptional ion-exchange properties are discussed in detail.

## 2 Experimental Section

***Caution!*** The UO_2_(NO_3_)_2_·6H_2_O used in this work contained natural uranium; nevertheless, the standard precautions for handling radioactive materials must be followed.

### 2.1 Materials and Methods

UO_2_(NO_3_)_2_·6H_2_O (International Bioanalytical Industries, Inc.), H_3_PO_3_ (Alfa-Aesar, 99.5%), CsOH·XH_2_O (X = 15–20%) (Sigma-Aldrich, 99%), and H_3_BO_3_ (Alfa-Aesar, 99.5%). NaNO_3_ (Alfa-Aesar, 99.5%), KNO_3_ (Alfa-Aesar, 99.5%) RbNO_3_ (Alfa-Aesar, 99.5%), Mg­(NO_3_)_2_·6H_2_O (Alfa-Aesar, 99.5%), Ca­(NO_3_)_2_·4H_2_O (Alfa-Aesar, 99.5%), SrCl_2_·6H_2_O (Alfa-Aesar, 99.5%), BaCl_2_·2H_2_O (Alfa-Aesar, 99.5%), NiCl_2_·6H_2_O (Alfa-Aesar, 99.5%), CoCl_2_·6H_2_O (Alfa-Aesar, 99.5%), Cu­(NO_3_)_2_·6H_2_O (Alfa-Aesar, 99.5%), Zn­(NO_3_)_2_·6­(H_2_O) (Alfa-Aesar, 99.5%), Cd­(NO_3_)_2_·6H_2_O (Alfa-Aesar, 99.5%), PbCl_2_ (Alfa-Aesar, 99.5%), Bi­(NO_3_)_3_ (Alfa-Aesar, 99.5%), [La–Lu­(NO_3_)_3_·6H_2_O] (Alfa-Aesar, 99.5%), and Th­(NO_3_)_4_·5H_2_O (International Bioanalytical Industries, Inc.).

#### 2.1.1 Synthesis of CUPB1

Compound CUPB1 was synthesized from the hydrothermal method with initial compositions of UO_2_(NO_3_)_2_·6H_2_O (0.0515 g, 0.10 mmol), CsOH·xH_2_O (0.0605 g, 0.40 mmol), H_3_PO_3_ (0.0332 g, 0.40 mmol), H_3_BO_3_ (0.0649 g, 1.05 mmol), and deionized water (0.5 mL) with a ratio of U:Cs:P:B = 1:4:4:10. All the chemicals were mixed thoroughly in an agate mortar and then sealed into a Teflon-lined stainless steel autoclave (23 mL). The autoclave was put into a box furnace and heated up to 220 °C for 36 h, and then cooled down to 50 °C at a cooling rate of 3 °C/h. Then, the furnace was switched off. The resulting products were washed with hot water and ethanol to get rid of the excessive boric acid. Yellow large block-shaped crystals CUPB1 together with a greenish byproduct crystals of Cs_2_(UO_2_)_2_(PO_4_)_2_ ^31^ were obtained. The yield of CUPB1 is as high as ca. 49% based on the U content. Pure phases of CUPB1 were obtained from picking up large crystals. The pure phase and ion-exchanged samples of CUPB1 were characterized and confirmed by using laboratory XRD (Figures S1 and S2). Energy-dispersive X-ray spectroscopy (EDS) elemental analyses on the crystals of CUPB1 yield a molar ratio result of U:Cs:P = 3.08:2.96:3.95, which is consistent with the composition obtained from single-crystal diffraction studies (Figure S3).

#### 2.1.2 Ion-Exchange Experiments of CUPB1

Ionic exchange reactions were performed by polycrystalline CUPB1 (50 mg) in scintillation vials with 10 mL of ∼0.005 M SrCl_2_(H_2_O)_6_ (aq), BaCl_2_(H_2_O)_2_ (aq), PbCl_2_ (aq), NiCl_2_(H_2_O)_6_ (aq), and CoCl_2_(H_2_O)_6_ (aq) solutions. The reactions were kept at room temperature for 2 h under magnetic stirring and then transferred into a box furnace at 70 °C for 12 h. The ion-exchanged samples were separated through filtration, washed with excess of water and acetone for 1 day, and then placed into a drying oven for 24 h. The compositions and element distributions of exchanged samples were measured by EDS measurements.

The kinetic studies of Sr^2+^, Ba^2+^, Pb^2+^, Co^2+^, and Ni^2+^ ion-exchanged by CUPB1 were carried out as follows. Nine parallel solutions (10 mL) for each cation (0.005 M) were mixed with the same ratios of *A* ^2+^:CUPB1 = 3:2 (*A* ^2+^:Cs^+^ = 1:2, 50 mg CUPB1 sample for each), and the mixtures were kept under magnetic stirring at room temperature (RT). The ion-exchange experiments were performed for nine different reaction times (1.5, 4, 21, 26, 45, 48, 52, 69, and 74 h). For comparison, the second series of experiments were performed in a box furnace at 70 °C with the same reaction times. The suspensions were filtered at certain reaction times, and the filtrates were analyzed by ICP-MS (Table S2). The final solid samples were analyzed by EDS and PXRD (Figures S3 and S4).

### 2.2 Crystallographic Studies and Powder X-ray Diffraction

Diffraction data for CUPB1 were collected on an Agilent Technologies SuperNova diffractometer with Mo–Kα radiation (λ = 0.71073 Å) at room temperature. All data sets were corrected for Lorentz and polarization factors as well as for absorption by the multiscan method.^32a^ Structure of compound CUPB1 was solved by direct method and refined by a full-matrix least-squares fitting on *F* ^2^ by SHELX-97.^32b^ Its structure was checked for possible missing symmetry elements using PLATON with the ADDSYM algorithm, and no higher symmetry was found.^32c^ Crystallographic data and structural refinements for CUPB1 are summarized in Table 1. More information on the important bond distances and angles of CUPB1 are listed in Table S1.

**Table 1:** Crystal Data and Structure Refinements for Compound CUPB1^a^

compound	CUPB1
FW	3215.02
Space group	*P*4_1_2_1_2
*a*(Å)	12.2376(3)
*b*(Å)	12.2376(3)
*c*(Å)	33.9468(11)
α(deg)	90
β(deg)	90
γ(deg)	90
V(Å^3^)	5083.8(2)
Z	4
λ(Å)	0.71073
F(000)	5488
*D*_C_(g cm^–3^)	4.201
GOOF on F^2^	1.073
R_1_	0.0304
*w*R_2_	0.0740

a *R*_1_ = ∑||*F*_*o*_| – |*F*_*c*_||/∑|*F*_*o*_|, *w*R_2_ = {∑*w­[*(*F*_*o*_)^2^ – (*F*_*c*_)^2^ *]* ^2^/∑*w* [(*F*_*o*_)^2^] ^2^}^1/2^

X-ray powder diffraction data were measured on a Bruker-AXS D4 Endeavor diffractometer, 40 kV/40 mA, in Bragg–Brentano geometry. The diffractometer is equipped with a copper X-ray tube and a primary nickel filter producing Cu Kα_1,2_ radiation (λ = 1.54187 Å). A linear silicon strip LynxEye detector (Bruker-AXS) was used. Data were recorded in the range of 2θ = 10–80 ° with 10 s/step and a step width of 0.02 °. The aperture of the fixed divergence slit was set to 0.2 mm, and the receiving slit was set to 8.0 mm, respectively. The discriminator of the detector was set to an interval of [0.16–0.25 V].

### 2.3 Scanning Electron Microscopy (SEM)/Energy-Dispersive X-ray Spectroscopy (EDS) Analyses

Elemental analyses, scanning electron microscopy image, and energy-dispersive X-ray spectroscopy (SEM/EDS) were collected on an FEI Quanta 200F environment scanning electron microscope with a low-vacuum mode at 0.6 mbar. SEM/EDS results are given in the Supporting Information (Figure S3).

### 2.4 Raman Spectroscopy

Unpolarized Raman spectra were recorded with a Horiba LabRAM HR spectrometer by using a Peltier cooled multichannel CCD detector. An objective lens with a 50× magnification was linked to the spectrometer, allowing the analysis of samples as small as 2 μm in diameter. The samples were in the form of crystals. The incident radiation was produced by a He–Ne laser line at a power of 17 mW (λ = 632.8 nm). The focal length of the spectrometer was 800 mm, and a 1800 gr/mm grating was used. The spectral resolution was approximately 1 cm^–1^ with a slit of 100 μm. The spectrum was recorded in the range of 100–4000 cm^–1^.

### 2.5 Thermal Analysis (TG-DSC Experiments)

The thermal behavior of the polycrystalline CUPB1 up to 1200 °C was studied by differential scanning calorimetry (DSC) analysis coupled with thermogravimetry (TG) in air at a heating rate of 10 °C/min using a Netzsch STA 449C Jupiter apparatus. The sample (12.5 mg) was loaded into a platinum crucible, which was covered with a platinum cover. During the measurements, a constant air flow of 20–30 mL/min was applied.

### 2.6 Bond-Valence Analysis

As a semiempirical method for the approximate determination of valence states, BVS of all atoms in phase CUPB1 were calculated and agree well with corresponding formal values of oxidation states. The bond-valence parameters for U­(VI)-O, Cs­(I)-O, P­(V), and B­(III)-O were used according to Brese and O’Keeffe.^33^ (Table S2)

### 2.7 Inductively Coupled Plasma Mass Spectrometry (ICP-MS) Analyses

ICP-MS is a type of mass spectrometry that is capable of detecting metals and several nonmetals at concentrations as low as one part in 10^15^ (part per quadrillion, ppq) on noninterfered low-background isotopes.^34^ The concentrations of metal ions in the solution before and after ion-exchange experiments were analyzed using PE Sciex ELAN 6100 ICP-MS instrumentation. All samples (including standards) were prepared in a 1% nitric acid solution. All of the ion-exchanged solution samples were diluted to lower the concentrations below 100 ppb for ICP-MS measurements.

## 3 Results and Discussion

### 3.1 Synthesis

The investigation of the Cs–U–P–B-O system, a microporous alkaline metal uranyl borophosphate CUPB1, was carried out in mild hydrothermal conditions. It was prepared by a very simple procedure with starting chemicals in a ratio of UO_2_(NO_3_)_2_(H_2_O)_6_:H_3_BO_3_:H_3_PO_3_:CsOH·x­(H_2_O) = 1:10:4:4, together with 0.5 mL of deionized water. For understanding the reaction mechanism more clearly, we have performed the following experiments with the different ratios of UO_2_(NO_3_)_2_(H_2_O)_6_:H_3_BO_3_:H_3_PO_3_:CsOH·x­(H_2_O) = 1:1:4:4; 1:3:4:4; 1:5:4:4; 1:8:4:4; 1:10:4:4; 1:12:4:4, and 1:15:4:4. The final results are that we can only get the crystals CUPB1 in the ratios of 1:8:4:4; 1:10:4:4 and 1:12:4:4, in which the highest yield is from the ratio of 1:10:4:4. The main byproducts of the above syntheses are the cesium uranyl phosphate, Cs_2_(UO_2_)_2_(PO_4_)_2_,^31^ which is a quite stable phase in this reaction system. Thus, we supposed that H_3_BO_3_ has played both important roles of flux and reaction medium in the hydrothermal reactions. Note that the first three actinide borophosphates were prepared with H_3_BO_3_–NH_4_H_2_PO_4_ flux method at around 200 °C with quite less water (50 μL).^29b^ Changing from H_3_PO_3_ to NH_4_H_2_PO_4_, the preparation of compound CUPB1 was unsuccessful with powder products, and no crystal phases formed, which indicated that the crystal CUPB1 formation process requires a lower pH value.

#### 3.2.1 Structure of CUPB1

The compound CUPB1 has a unique microporous structure that crystallizes in the chiral space group of *P*4_1_2_1_2 (No. 94). There are three Cs, three U, one B, and four P atom positions in the asymmetric unit. CUPB1 is an extremely rare porous borophosphate, which is built from three basic building units (BBU), UO_7_ pentagonal bipyramids, BO_4_, and PO_4_ tetrahedra.^35–37^ One BO_4_ tetrahedron exhibits corner sharing with four PO_4_ tetrahedra, forming the [B­(PO_4_)_4_] clusters. From the fundamental building blocks (FBBs) view, the [B­(PO_4_)_4_] can also be described as (5□:[□]□|□|□|□|) according to the borate classification system.^10^ As shown in Figure S4c, the [B­(PO_4_)_4_] FBBs are isolated inside its 3D framework structure, which is similar to the actinide-free borophosphate Na_3_Cd_3_B­(PO_4_)_4_ and Cs_2_Cr_3_(BP_4_O_14_)­(P_4_O_13_).^38a,38b^ The U(2)­O_7_ and U(3)­O_7_ pentagonal bipyramids are corner or edge sharing with those [B­(PO_4_)_4_] FBBs forming two mirror symmetric S-type uranyl borophosphate chains, [U(2)­O_2_]­[B­(PO_4_)_4_] and [U(3)­O_2_]­[B­(PO_4_)_4_] (UBP-chains), along the *a*-axis (Figure 1a,b). The two S-type UBP-chains bridge vertex sharing linked by [B­(PO_4_)_4_] anionic groups, forming a 2D uranyl borophosphate sheet [U(2)-BP_4_–U­(3)] on the *ab*-plane, in which 8-Rings (8-Rs) with four repeated linkages -UO_7_–PO_4_–, can be observed along the *c*-axis (Figure 1c). They paralleled U(2)-BP_4_–U­(3) layers packing in a mode of ---*ABCDA*--- with a periodic time of four. U(1)­O_7_ pentagonal bipyramids acted as the linkers further connected the paralleled U(2)-BP_4_–U­(3) layers along the *c*-axis, constructed its 3D porous uranyl borophosphate framework [(UO_2_)_3_B­(PO_4_)_4_]^3–^ (Figure 2a,b). Three different multi-intersection 8-R tunnels, running along the [001], [110], and [−110] directions, can be observed in the anionic network. Cs^+^ cations are disordered and located in the tunnels and voids of the framework for charge balance (Figure 2c).

**Figure 1:**
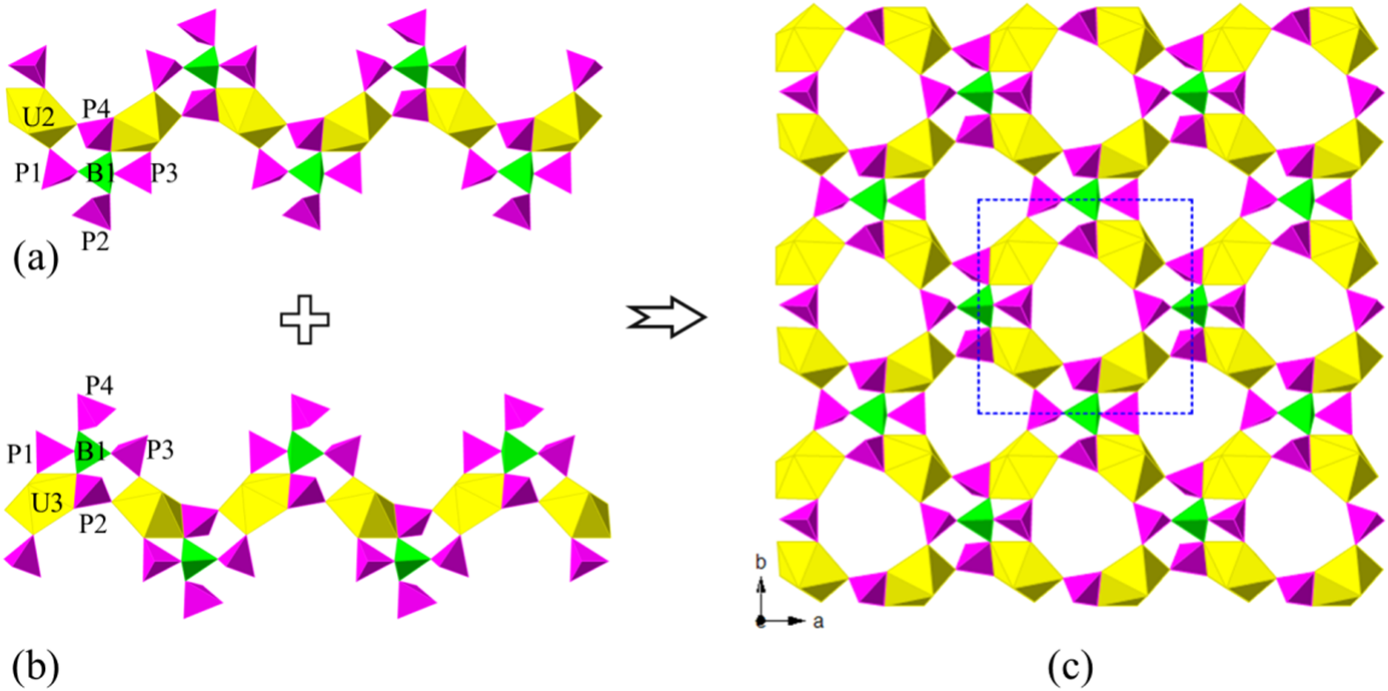
Zigzag uranyl borophosphate chain U(2)­B­(PO_4_)_4_ (a) and U(3)­B­(PO_4_)_4_ (b) along the *a*-axis; a 2D uranyl borophosphate sheet on the *ab*-plane (c). UO_7_ polyhedra, BO_4_, and PO_4_ tetrahedra are shown in yellow, green, and pink, respectively.

**Figure 2:**
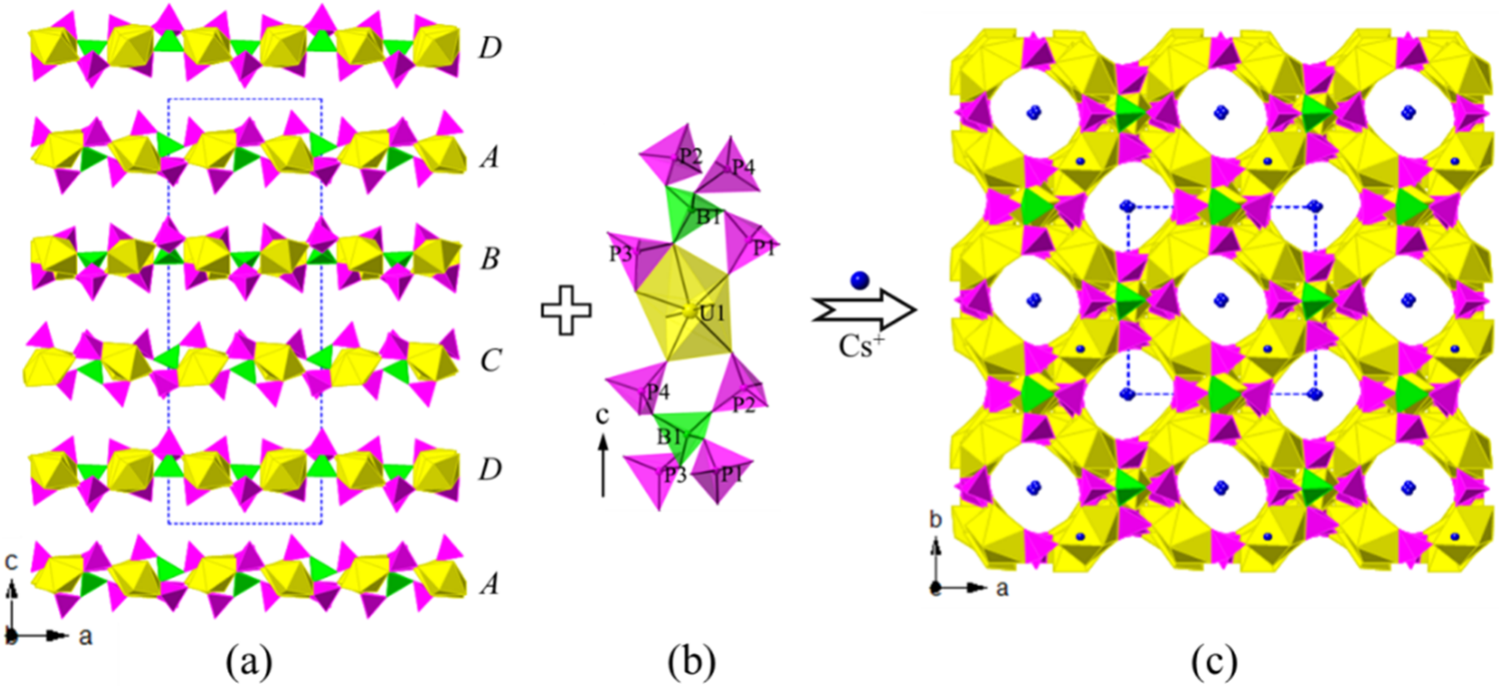
2D uranyl borophosphate sheets [U­(2/3)­B­(PO_4_)_4_] are parallel arranged along the *c*-axis (a); 1D [U(1)­B­(PO_4_)_4_] chain along the *c*-axis (b); and view of the 3D framework structure of CUPB1 down the *c*-axis (c). UO_7_ polyhedra, BO_4_, PO_4_ tetrahedra, and Cs atoms are shown in yellow, green, pink, and blue, respectively.

The B–O bond lengths in the distorted BO_4_ tetrahedron range from 1.38(2) to 1.57(2) Å, and the O–B–O bond angles are in the range of 104.0(10)°–117.1(11)°. For PO_4_ tetrahedra, the P–O bond distances are in the range of [1.493(9)–1.604(9) Å], while the O–P–O bond angles range from 99.5(5)° to 113.3(5)°, which are consistent with those of borophosphates reported previously.^2^ The axial U–O bond lengths for U(1)­O_7_ pentagonal bipyramids are 1.758(10) and 1.768(12) Å, whereas its equatorial U–O bond distances are in the range of [2.284(11)–2.648(9) Å]. The axial U(2)-O bond lengths for U(2)­O_7_ polyhedra are 1.776(9) and 1.775(9) Å, and the equatorial U(2)-O bond distances range from 2.284(8) to 2.648(8) Å. For U(3)­O_7_ pentagonal bipyramids, the axial U(3)-O bond lengths are 1.768(9) Å and 1.781(9) Å, the equatorial U(3)-O bond distances are in the range of [2.274(9)–2.683(8) Å], which are in good agreement with uranyl borates reported earlier^29b,29c^ (Table S1). The calculated BVS values for B(1), P(1)–P(4), and U(1)–U(3) are ca. 3.04, 5.15, 5.18, 5.21, 5.06, 6.08, 5.99, and 6.0, respectively, which confirmed the valence states of B, P, and U are 3+, 5+, and 6+ in CUPB1. The Cs^+^ cations are 10-, 11-, and 12-fold oxygen coordinated, with Cs–O bond lengths from 3.189(9) Å to 3.81(4) Å (Tables S1 and S2)

The key feature of CUPB1 is the nanoscale size cages, U_12_P_24_B_8_, which are built from 12 UO_7_ pentagonal bipyramids, 24 PO_4_ tetrahedra, as well as eight BO_4_ tetrahedra. Its diameters are ∼12.2 Å × 12.2 Å × 11.7 Å and are in a volume of ∼1741.4 Å^3^. It is the first example of such a large uranyl-based cage containing both PO_4_ and BO_4_ tetrahedra. The complex U_12_P_24_B_8_ cage has six 8-Rs windows in the six directions of its faces, further bridging connected with six other neighboring ones (Figure 4b). Six Cs^+^ cations are located near the windows inside the U_12_P_24_B_8_ cage in a square bipyramid geometry. Compared with those uranyl peroxide cage clusters reported by Burns et al.,^39^ the incorporation of BO_4_ tetrahedra makes the U_12_P_24_B_8_ cage a more complex topology. Not like the uranyl groups edge sharing with each other to build its hemisphere or even the whole skeleton in U_28_ or U_124_P_32_ cages,^40^ uranyl groups are isolated in the U_12_P_24_B_8_ cages. U(2) and U(3) pentagonal bipyramids linked by [B­(PO_4_)_4_] FBBs, forming the hemisphere, U_4_B_4_P_12_, of the cage; four U(3) ions in the equatorial line bridge connected with the two symmetric U_4_B_4_P_12_ hemispheres, creating the unusual nanoscale size uranyl borophosphate cage, U_12_P_24_B_8_ (Figure 3).

**Figure 3:**
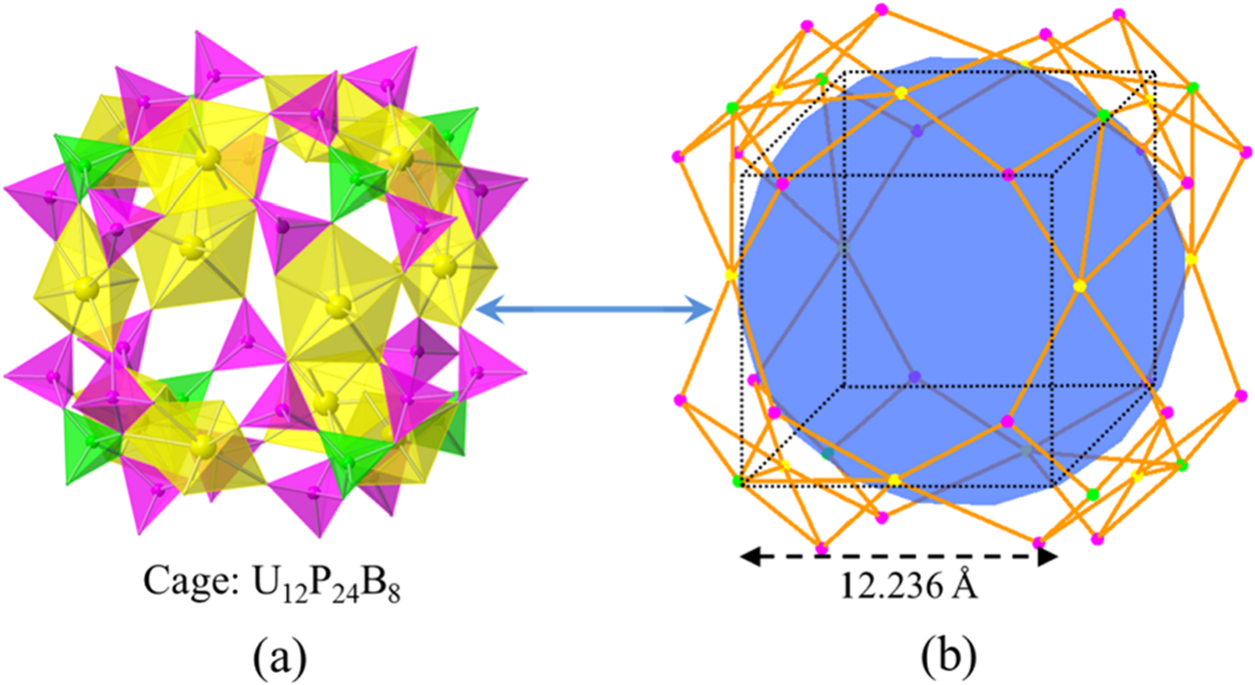
A spherical uranyl borophosphate cage [U_2_P_24_B_8_] polyhedral (a) and topology (b) representation. UO_7_ polyhedra, BO_4_, PO_4_ tetrahedra, and Cs atoms are shown in yellow, green, pink, and blue, respectively.

#### 3.2.2 Framework Topology Characterization

The 3D anionic framework of CUPB1 is extremely complex and difficult to describe clearly. In order to give a detailed description of the uncommon zeolite-like topology network of CUPB1, the simplified anionic framework [(UO_2_)_3_B­(PO_4_)_4_] of CUPB1 was plotted through omitting the oxygen anions (Figure S5). The simplified cationic net of CUPB1 can be described as a new 8-nodal net topological type with a Schläfli symbol of {3.4^2^.5^2^.6}_3_{3^2^.4^2^.8^3^.9^3^}_3_{3^3^.4^3^}­{3^6^.4^9^.5^6^}^41^ (Figure 4a,b). The 3D porous framework of CUPB1 has a low framework density of ∼12.6 M atoms (M is the cations of the framework) per 1000 Å^3^, which is even lower than the open zeolite faujasite with framework density of ∼13.5.^42a^ Each of the UO_7_ pentagonal bipyramid is surrounded by two [B­(PO_4_)_4_] FBBs, whereas six UO_7_ pentagonal bipyramids are sitting around each [B­(PO_4_)_4_] FBB unit (note as *X* here), existing as a *X*U_6_ square bipyramid geometry. Thus, the ratio of *X* and U is 1:3 in a *X*U_3_ network structure (Figure 5). If we simplified the porous anionic framework [(UO_2_)_3_B­(PO_4_)_4_]^3–^ of CUPB1 with FBB [B­(PO_4_)_4_] as a 6-connected node (*X*) in the 3D network, we can get a *X*U_3_ topology network as shown in Figure S6a,b,c; this simplified cationic net is a new 2-nodal net topological type with the Schläfli symbol of {8^12^.12^3^}­{8}_3_ ^41^ (Figure S6).

**Figure 4:**
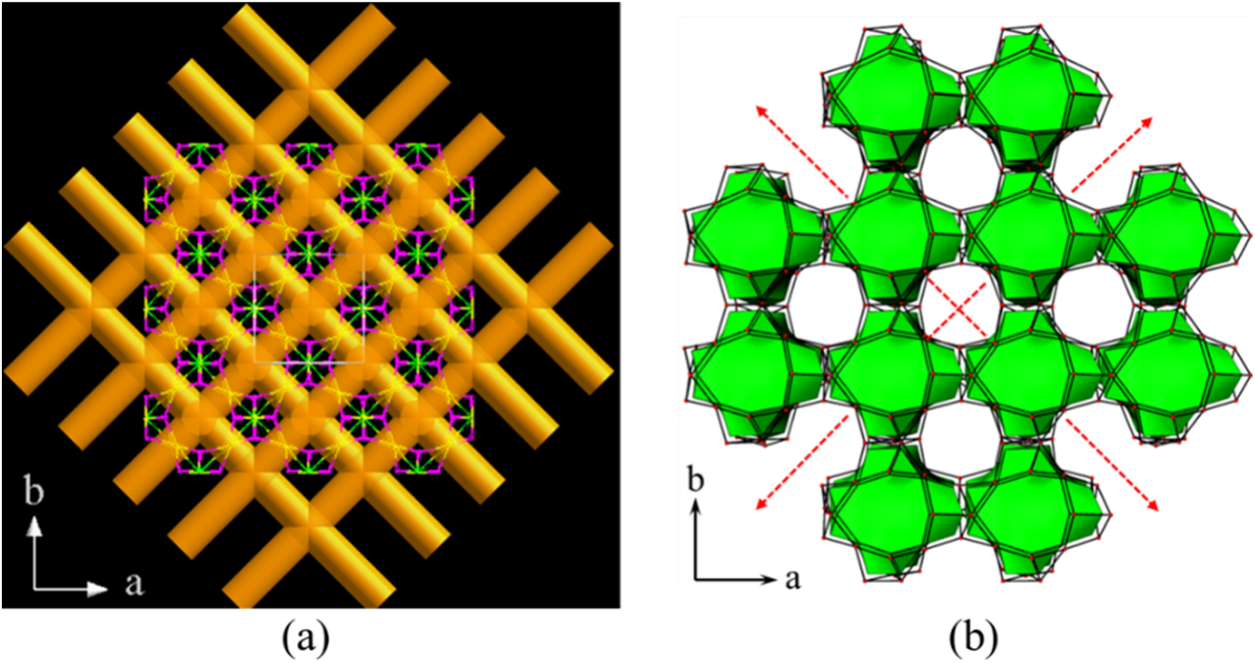
Tracing of the multi-intersection tunnels within the 3D framework of CUPB1 with stick fashion (a) and the [U_2_P_24_B_8_]-cages packing mode (b).

**Figure 5:**
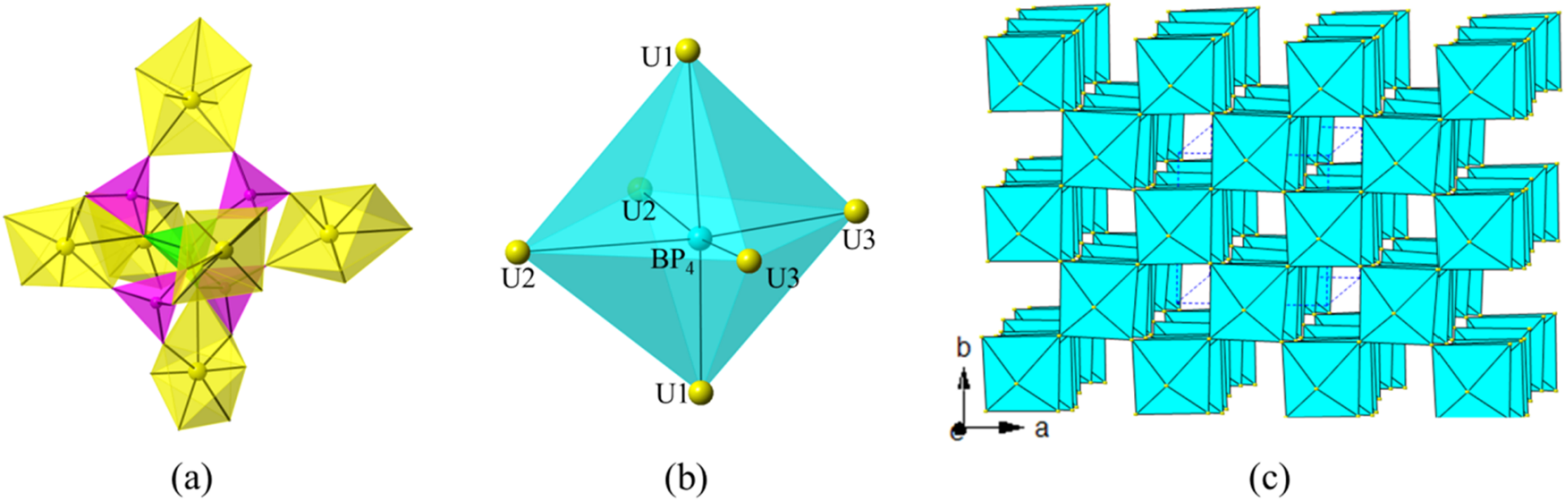
Primary uranyl borophosphate cluster [(UO_2_)_6_B­(PO_4_)_4_] with B-center (a); a simplified topology of cluster [(UO_2_)_6_B­(PO_4_)_4_] (square bipyramid geometry) (b); view of the simplified topology representation of CUPB1 down the *c*-axis (c).

The channel system and cavities of CUPB1 can be better illustrated by natural tiling by tracing the colors of the tiles. From the natural tiling point of view, the anionic uranyl borophosphate framework of CUPB1 is built from four different tiles, in which the primary build unit (PBU) is the cage [7^4^·8^2^]. These [7^4^·8^2^] PBUs connected with four other neighboring ones on the *ab*-plane; leave a large hole surrounded by four [7^4^·8^2^] cages. These large cavities were filled with another nanosize cage [3^8^·4^12^·8^8^] bridged by the tile of [3^4^·8^2^], defining a 2D layer parallel with the ab-plane. Those paralleled 2D layers were further linked by a [3^2^·8^2^] tile, forming its extraordinary 3D tiling frameworks. From the tiling model of CUPB1, it is easier to trace its tunnels, cages, and cavities with tiling signatures of [7^4^·8^2^] + [3^8^·4^12^·8^8^] + [3^4^·8^2^] + [3^2^·8^2^] (Figures 6 and S6).

**Figure 6:**
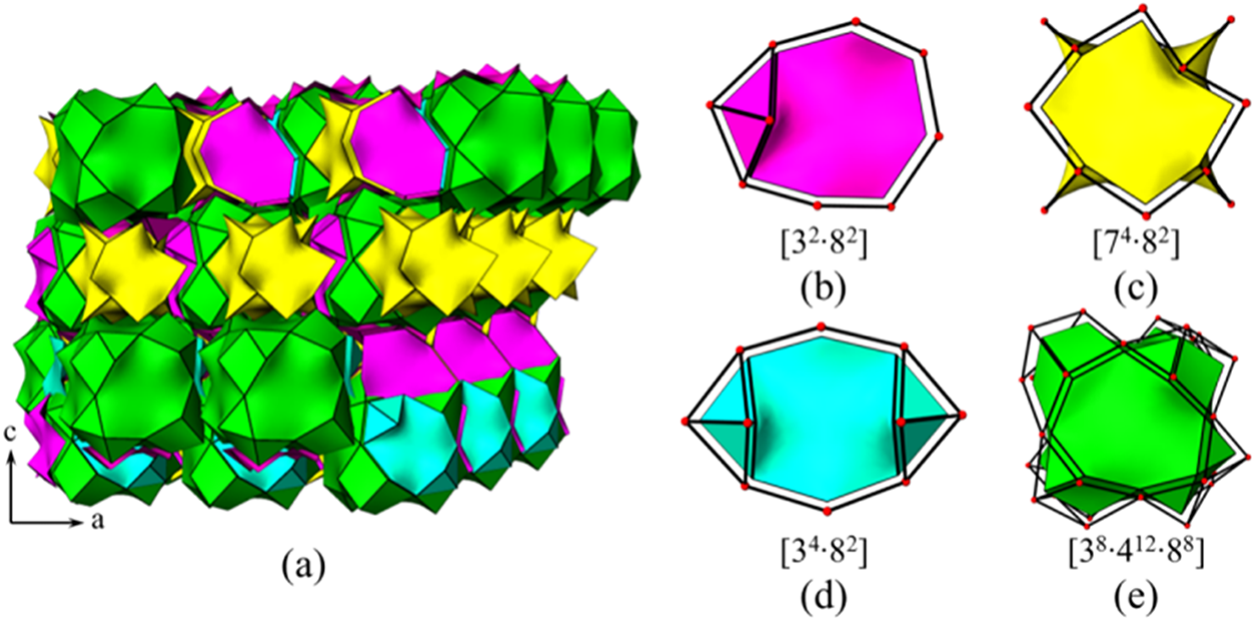
View of the 3D framework structure of CUPB1 using tiling (a) and the basic tiles of its tiling structure [3^2^·8^2^] (b), [7^4^·8^2^] (c), [3^4^·8^2^] (d), and [3^8^·4^12^·8^8^] (e).

#### 3.3.1 Ion-Exchange Properties

CUPB1 is an exceedingly porous material with a high free void volume within its 3D open framework structures. Furthermore, the Cs^+^ ions are disordered and reside in the free voids of the porous network; these have prompted us to investigate their ion-exchange properties.^43,44^ Ion-exchange experiments were conducted with a variety of cations, from monovalent *A* ^+^ to tetravalent *A* ^4+^ at room temperature and elevated temperatures. CUPB1 has shown significant ion-exchange properties with *A* ^2+^. The ion-exchanged samples were further studied by EDS elemental analyses, element distribution mapping, ICP-MS analyses, as well as powder X-ray diffraction (PXRD).^45^ From the PXRD patterns, we can see that the framework structure of CUPB1 remains intact after the ion-exchange experiments, which has the same characteristics as the reported zeolites and MOF materials^3^ (Figures S2 and S3, Table S2). Compared with porous zeolites and MOFs reported in the literature, we note that while zeolites^4^ and MOFs^5,6^ often have higher capacities due to their microporosity, CUPB1 offers distinct advantages: (1) exceptional selectivity for large, hard cations like Sr^2+^, Ni^2+^, etc., and (2) high chemical and potential radiation stability inherent to inorganic uranium phases. This positions CUPB1 as a specialized ion exchanger for challenging environments, where stability is paramount.

#### 3.3.2 Kinetic Studies of the Cations A^2+^ (A = Sr, Ba, Pb, Co, Ni) Exchange

Sorbents are particularly desirable, since they can adsorb the very toxic ions, such as Pb^2+^, Ni^2+^, Co^2+^, etc.,^42b,42c^ and some radionuclides, such as ^137^Cs, ^90^Sr, ^137m^Ba, etc.^42d,42e^ Because CUPB1 has promising ion-exchange results as described above, we explored in more detail the toxic cation remediation properties. The kinetics of Sr^2+^ ion-exchange was investigated, and it was shown that the concentrations of Sr^2+^ (∼450 ppm at a V/m ratio of 200 mL/g) decreased rapidly: the relative amount of Sr-removed reached ∼56% after 24 h and increased to ∼70.2% after 74 h at room temperature (Figure 9a and Table S2). Based on eq 1, we can calculate the Sr-exchange capacity *q* ^Sr^ is ∼64.1 mg/g. The distribution coefficient *K*_*d*_ is a measurement of affinity and selectivity shown as eq 2. The Sr-exchanged *K*_*d*_^Sr^ at RT for CUPB1 is 493.3 mL/g. It is interesting to note that, when the temperature increases to 70 °C, both the *q* ^Sr^ and *K*_*d*_^Sr^ have improved to 74.2 mg/g and 942.8 mL/g, respectively (Figure 9a and Table S2). The Sr-removed amount *R* increased up to 82.9% after 72 h, which is comparable with the Sr-exchangers reported previously.^46^

We argue that temperature is one of the important driving forces for ion-exchange reactions. Similarly, we have performed Ba-exchange experiments with Sr^2+^ in the same conditions.^47,48^ The calculated Ba-exchange capacity of *q* ^Ba^ is ∼100.4 mg/g and *K*_*d*_^Ba^ of ∼498.6 mL/g based on eqs 1, 2, and 3 at RT.^49^ As expected, both the *q* ^Ba^ and *K*_*d*_^Ba^ improved to ∼113.4 mg/g and ∼830.3 mL/g at 70 °C, and the Ba-removed amount approached ∼80.7% (Figure 9b and Table S2). For comparison, the relative Sr-exchange properties of CUPB1 are better than the Ba ones. We assumed that because the radius of Sr^2+^ is smaller than that of Ba^2+^ cations, the Sr-exchange mechanism needs a lower adsorption energy.^33^ The ionic radii for cations are Cs^+^ (∼1.69 Å), Sr^2+^ (∼1.26 Å), and Ba^2+^(∼1.42 Å). While Ba^2+^ is smaller than Cs^+^, its size is still relatively larger than that of Sr^2+^ for the channels in CUPB1. More importantly, the driving force for exchange is a combination of hydration energy and lattice energy. Sr^2+^ has a charge density much higher than that of Ba^2+^, leading to a more negative hydration energy. This makes the dehydration of Sr^2+^ (a necessary step for entering the crystal lattice) less favorable, but once inside, its higher charge provides a stronger electrostatic interaction with the anionic framework, which likely dominates the thermodynamics for this system. The larger Ba^2+^ may experience greater steric hindrance within the channels, slowing its diffusion and reducing the overall exchange capacity. We presume that the optimal fit and stronger Coulombic attraction for Sr^2+^ likely explain its higher exchange rate.

1 $$q=\frac{{(C}_{0}-C_{e})V}{m}$$

2 $$K_{d}=\frac{V}{m}\frac{\left(C_{0}-C_{f}\right)}{C_{f}}$$

3 $$R=\frac{\left(C_{0}{-C}_{f}\right)}{C_{0}}$$

In eqs 1, 2, and 3, *C*_0_ and *C*_*f*_ represent the initial and equilibrium concentrations of the ions as measured by ICP-MS, *V* is the solution volume, *m* is the mass of CUPB1, and *R* is the relative amount of cation removed.

Heavy metal ions in solution are toxic to humans if the concentration is sufficiently high. For example, Pb is a highly poisonous metal (whether inhaled or swallowed), affecting almost every organ and system in the human body.^50a^ Therefore, during the last decades, how to separate the toxic ions from the solutions efficiently is a one of the core questions.^50^ Removal of inorganic pollutants from the aqua can be achieved by electrodialysis,^50b^ chemical precipitation,^50c^ adsorption,^50d^ solvent extraction,^50e^ and ultrafiltration^50f^ or ion exchange.^50g^ CUPB1 shows a promising application for ion exchange with Pb^2+^ from Pb-contaminated solutions. The kinetics of Pb^2+^ ion-exchange showed that the Pb amount (∼950 ppm at a V/m ratio of 200 mL/g) decreased ∼76.9% after 72 h at RT. The Pb-removed amount has increased to 83.6% at the temperature of 70 °C (Figure 9c and Table S2). The Pb-exchange capacity of *q* ^Pb^ is ∼146.4 mg/g at RT and ∼169.4 mg/g at 70 °C. The *K*_*d*_^Pb^ at RT is 720 mL/g, whereas the higher *K*_*d*_^Pb^ value reached 1640 mL/g at 70 °C (Figure 9c and Table S2). The promising Pb-exchanged properties of CUPB1 are comparable with those previously reported modified zeolites^34^ ^b,c^ and resins (Figure 7).^33^

**Figure 7:**
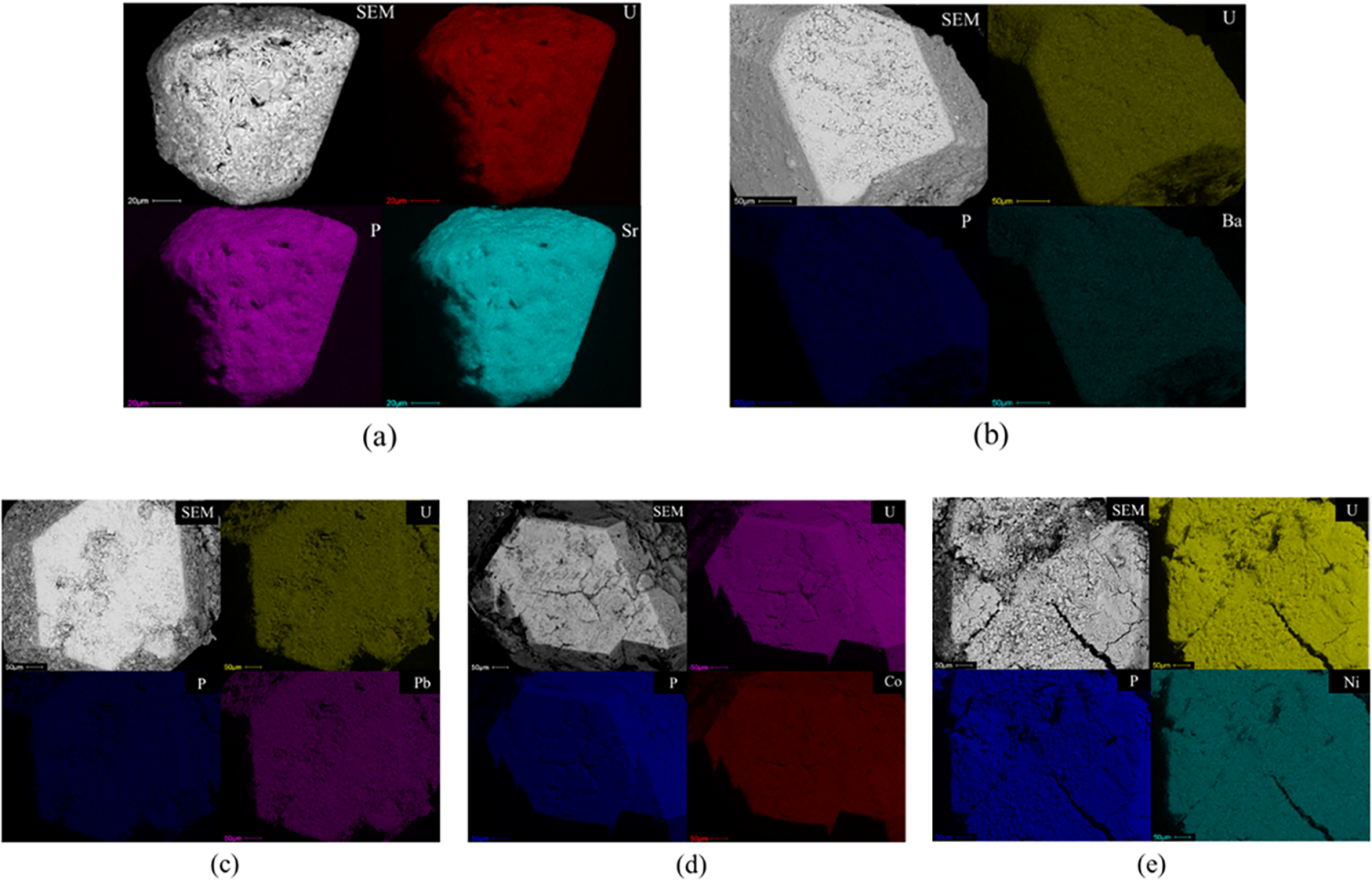
SEM image of the Sr (a), Ba (b), Pb (c), Co (d), and Ni (e)-exchanged samples and elemental distribution maps.

There are groups of divalent transition-metal cations (Co^2+^, Ni^2+^, Zn^2+^, Cu^2+^, *etc*.) exchangers that have been synthesized and characterized, such as resins,^51a^ MOFs,^51b^ zeolites or zeolite-like materials,^51c^ and polymers.^51d^ Among them, MOFs, zeolites, or zeolite-like materials preserve their original crystal structure after ion-exchange reactions, which resembles CUPB1.^52–54^ As shown in Figure 8, the CUPB1 crystals show the colors of the transition metals (Co^2+^ and Ni^2+^) within a few days. The crystals can be cut, and the interior shows the same color as the surface. EDS, elemental distribution maps, and ICP-MS data were collected from crystals and the solutions after exchange, and these can demonstrate the presence of the cations within the crystals (Figure S3 in the Supporting Information).^55,56^ CUPB1 exhibits a better Co^2+^ adsorption at a higher temperature of 70 °C (*R*∼74% after 72 h) compared to the RT (*R*∼64%). Accordingly, the exchange capacity *q* ^Co^ is ∼44.4 mg/g at 70 °C, higher than that at ∼38.6 mg/g at RT. The distribution coefficient *K*_*d*_^Co^ increased to 569.2 mL/g (70 °C) from 355.6 mL/g (RT) (Figure 9d and Table S2). The maximum Ni^2+^ exchange capacity *q* ^Ni^ of CUPB1 was found to be ∼40.6 mg/g at RT, the corresponding removed amount is ∼67.7% and the *K*_*d*_^Ni^ is ∼ 425.1 mL/g. When the exchanged temperature increased up to 70 °C, the *q* ^Ni^ and *K*_*d*_^Ni^ can be as high as ∼45.4 mg/g and ∼633.3 mL/g, respectively (Figure 9e and Table S2).

**Figure 8:**
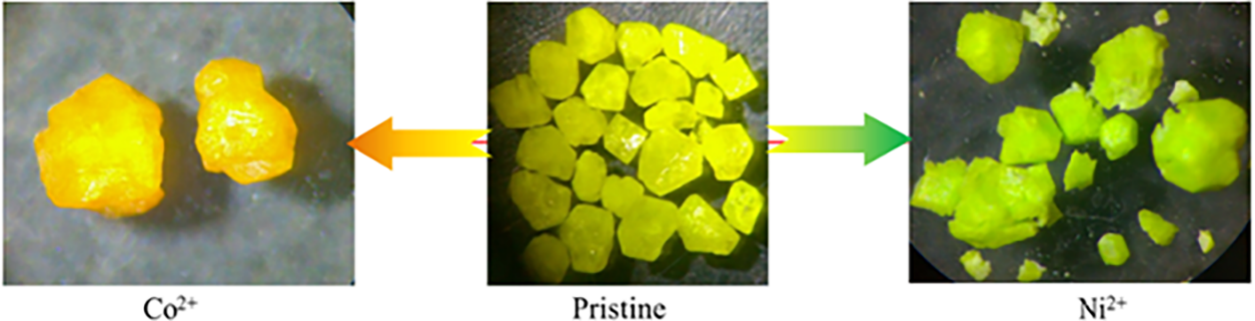
Photos of CUPB1 crystals before and after colored ions (Co^2+^ and Ni^2+^) exchanged samples.

**Figure 9:**
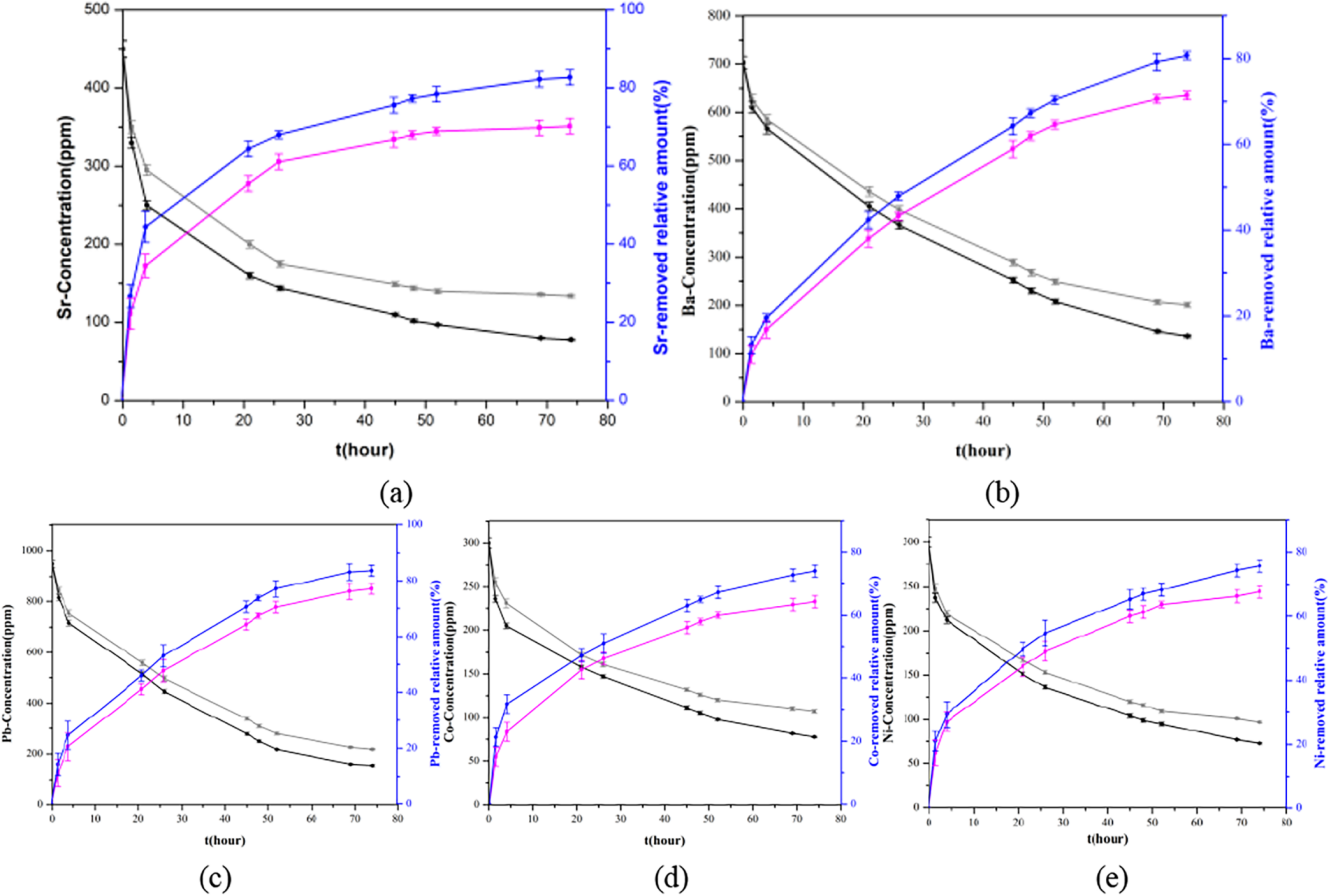
Kinetics of the *A* ^2+^ (Sr^2+^/Ba^2+^/Pb^2+^/Co^2+^/Ni^2+^) ion-exchanged with 50 mg of CUPB1 (*A*: Cs = 1:2) plotted as the *A*-concentration (ppm) (black/gray line) and the relative *A*-removed amount (%) (blue/pink line) vs time *t* (hour), respectively, (a–e). Pink and gray represent reaction at RT, and black and blue are at 70 °C.

### 3.4 Thermal Analysis

Thermogravimetric (TG) and differential scanning calorimetry (DSC) measurements were performed from 50 to 1200 °C (Figure S9). TG analysis indicates that CUPB1 shows no obvious weight loss until 1000 °C.^57–59^ There is a small endothermic peak at 387 °C that can be assigned to the removal of 0.5 mol of water molecules per formula unit, which is too light to be shown in the TG curve. The endothermic peak at 835 °C is matched with the decomposition of the anhydrate phases. From the thermal behavior of CUPB1, we concluded that its porous framework is robust and stable.

### 3.5 Raman Analysis

The Raman spectra of CUPB1 and its ionic exchange products were measured in a range of 100–4000 cm^–1^, and for convenience, we have divided the spectra into two sections: a low-frequency part in 100–1300 cm^–1^ and a high-frequency region in 3000–4000 cm^–1^ (Figure S10). After comparison, the Raman spectra of CUPB1 and the ion-exchanged samples show no obvious difference, which confirms that the 3D uranyl borate framework is intact after ion-exchange experiments. More scattering peaks are in the range of 100–1000 cm^–1^, which is dominated by contributions from the (UO_2_)^2+^, BO_3_ triangles and BO_4_, PO_4_ tetrahedra modes of CUPB1. The Raman bands located in lower frequencies in the range of 190–300 cm^–1^ could be attributed to the uranyl ion with a υ_2_ bending mode.^60,61^ Raman bands with a series of peaks around 476 cm^–1^ could be assigned to O–B–O doubly degenerated symmetric bending ν_2_ mode in BO_4_ tetrahedra. The Raman peak at ∼642 cm^–1^ is attributed to the bending character *v*_4_ of BO_4_. Raman bands from 800 to 870 cm^–1^ come from the symmetric vibration υ_1_ mode of the uranyl (UO_2_)^2+^ units. The characteristic vibrations of uranyl (UO_2_)^2+^ υ_3_ antisymmetrical stretch mode are at bands of 936 cm^–1^ and 978 cm^–1^. The Raman bands at 1002 cm^–1^ and 1018 cm^–1^ can be attributed to the ν_1_ PO_4_ symmetric stretching and ν_3_ PO_4_ antisymmetric stretching modes. The Raman bands within 1100–1200 cm^–1^ can be attributed to the O–B–O triply degenerated asymmetric mode stretching ν_3_ mode in BO_4_ tetrahedra. These assignments are consistent with previously reported works.^62^

## 4 Conclusions

A remarkable ion exchanger CUPB1 was obtained from the mild hydrothermal method. Its microporous framework is based on the FBBs of the [B­(PO_4_)_4_]^9–^ and UO_7_ pentagonal bipyramids. The unusual nanoscale size (∼12.2 × 12.2 × 11.7 Å) of uranyl borophosphate cages, U_12_P_24_B_8_, is observed in the network structure. In its 3D open framework, there are three multi-intersection 8-R tunnels along the [001], [110], and [-110] directions. The disordered Cs^+^ cations reside in the tunnels and voids of the framework. Its free void volume is as high as ∼59%, which demonstrates it is an extremely high porous actinide compound. From the topological viewpoint, the 3D porous framework of CUPB1 has a very low framework density of ∼12.6 M atoms per 1000 Å^3^, which is comparable with the typical zeolite materials. Importantly, as a highly porous material, CUPB1 can adsorb the cations from monovalent to divalent in solution at room or elevated temperatures. The remarkable ion-exchange properties of CUPB1 can be attributed to its open framework with three-dimensional chiral channels and disordered guest cations. We have studied the ion-exchange properties of CUPB1 with some toxic metal cations, Pb^2+^, Co^2+^, and Ni^2+^ and important nuclear fission products of Sr^2+^ and Ba^2+^, at both room temperature and 70 °C. This has proved that CUPB1 is a promising potential ion exchanger for the separation or purification of the toxic cations and nuclear wastes contaminated waters. The ion-exchange properties of CUPB1 have improved a certain point for all the cation-exchanged experiments at a higher temperature of 70 °C. At RT, the ion-exchange capacity *q* for Sr^2+^, Ba^2+^, Pb^2+^, Co^2+^, and Ni^2+^ is ∼64.1, ∼100.4, ∼146.4, ∼38.6, and ∼40.6 mg/g, respectively. When the temperature increased to 70 °C, the *q* improved to ∼74.2, ∼113.4, ∼169.4, ∼44.4, and ∼45.4 mg/g. This study has demonstrated the excellent thermal stability of the CUPB1 framework structure. Further investigations of the monovalent cations, such as Li^+^, Na^+^, K^+^, Rb^+^, NH_4_^+^, etc., and uranyl borophosphate analogues of CUPB1 are underway.

## Supplementary Material



## Notes

Selected bond lengths and angles, topological figures, PXRD patterns, SEM-EDS measurements, ICP-MS data, TG-DSC curves, and Raman spectra (PDF)
