# Supplementary material for: Enhanced Ion-Exchange Properties of a Complex Microporous Uranyl Borophosphate
Source: Inorg Chem. 2025 Dec 9;64(50):24754–65. doi: 10.1021/acs.inorgchem.5c04481 (PMC12728919; doi:10.1021/acs.inorgchem.5c04481)
Supplement: Supplementary file 1 [file ic5c04481_si_001.pdf]

Supporting Information

Enhanced Ionic Exchange Properties of a Complex Microporous Uranyl Borophosphate

Yucheng Hao[a,*], Jingli Xu[a], Thomas E. Albrecht[b], Shuao Wang[c], Rüdiger-A. Eichel,[d] and Evgeny V. Alekseev[d,*]

[a.]School of Energy Materials and Chemical Engineering, Hefei University, Hefei 230000, China.E-mail: haoyco@hfuu.edu.cn.

[b.]Department of Chemistry and Nuclear Science and Engineering Center, Colorado School of Mines, Golden, Colorado, 80401 USA

[c.]School for Radiological and Interdisciplinary Sciences (RAD-X) and Collaborative Innovation Center of Radiation Medicine of Jiangsu Higher Education Institutions, Jiangsu 215123, China

[d.]Institute of Energy Technologies (IET-1), Forschungszentrum Jülich GmbH, 52428 Jülich, Germany E-Mail: e.alekseev@fz-juelich.de.

Table S1. Selected Important Bond Lengths (angstroms) for **CUPB1**.

P(1)-O(5)	1.505(10)	P(1)-O(6)	1.521(11)		
P(1)-O(18)	1.551(11)	P(1)-O(12)	1.519(11)	P(1)-O average	1.524
P(2)-O(1)	1.498(11)	P(2)-O(13)	1.504(11)		
P(2)-O(2)	1.506(11)	P(2)-O(14)	1.585(10)	P(2)-O average	1.523
P(3)-O(21)	1.509(11)	P(3)-O(16)	1.584(10)		
P(3)-O(11)	1.493(11)	P(3)-O(19)	1.501(11)	P(3)-O average	1.521
P(4)-O(22)	1.503(11)	P(4)-O(17)	1.503(11)		
P(4)-O(8)	1.604(11)	P(4)-O(15)	1.518(11)	P(4)-O average	1.532
B(1)-O(18)	1.38(2)	B(1)-O(16)	1.47(2)		
B(1)-O(14)	1.57(2)	B(1)-O(8)#11	1.491(19)	B(1)-O average	1.48
U(1)-O(20)	1.758(10)	U(1)-O(10)	1.768(12)	U(1)-O(13)#1	2.284(11)
U(1)-O(12)	2.291(11)	U(1)-O(22)	2.307(10)	U(1)-O(21)	2.402(10)
U(1)-O(16)	2.648(9)				

U(2)-O(4)	1.776(9)	U(2)-O(7)	1.775(9)	U(2)-O(11)#1	2.321(8)
U(2)-O(17)#4	2.284(8)	U(2)-O(5)#5	2.294(8)		
U(2)-O(15)	2.393(8)	U(2)-O(8)	2.648(8)		
U(3)-O(3)	1.768(9)	U(3)-O(1)#8	2.274(9)	U(3)-O(2)	2.384(8)
U(3)-O(9)	1.781(9)	U(3)-O(14)	2.683(8)		
U(3)-O(19)	2.317(9)	U(3)-O(6)#8	2.301(8)		
Cs(1)-O(2)	3.246(9)	Cs(1)-O(4)#9	3.274(9)	Cs(1)-O(6)#8	3.310(9)
Cs(1)-O(9)	3.316(9)	Cs(1)-O(19)#3	3.336(10)	Cs(1)-OW1#9	3.36(4)
Cs(1)-O(7)#6	3.362(9)	Cs(1)-O(11)#3	3.401(10)	Cs(1)-O(17)#9	3.469(10)
Cs(1)-O(15)#9	3.516(10)	Cs(1)-O(3)#3	3.705(9)	Cs(2)-O(7)	3.189(9)
Cs(2)-O(5)#5	3.230(8)	Cs(2)-O(13)#8	3.265(10)	Cs(2)-O(20)#5	3.270(10)
Cs(2)-O(10)	3.270(9)	Cs(2)-O(9)	3.283(9)	Cs(2)-O(21)	3.293(10)
Cs(2)-O(19)	3.462(10)	Cs(2)-O(15)	3.506(9)	Cs(2)-O(1)#8	3.615(10)
Cs(3)-O(20)	3.255(10)	Cs(3)-O(20)	3.255(10)	Cs(3)-O(22)	3.391(9)
Cs(3)-O(22)	3.391(9)	Cs(3)-O(21)#10	3.401(10)	Cs(3)-O(21)	3.401(10)
Cs(3)-O(4)#11	3.493(9)	Cs(3)-O(4)#12	3.493(9)	Cs(3)-O(11)#10	3.493(10)
Cs(3)-O(11)	3.493(10)	Cs(4)-O(12)#11	3.191(9)	Cs(4)-O(12)#8	3.191(9)
Cs(4)-O(2)	3.270(9)	Cs(4)-O(2)#13	3.270(9)	Cs(4)-OW2#8	3.28(6)
Cs(4)-OW2#11	3.28(6)	Cs(4)-O(3)	3.283(10)	Cs(4)-O(3)#13	3.283(10)
Cs(4)-O(6)#11	3.508(9)	Cs(4)-O(6)#8	3.508(9)	Cs(4)-OW1#3	3.81(4)
Cs(4)-OW1#9	3.81(4)				

O(20)-U(1)-O(10)	178.2(5)	O(7)-U(2)-O(4)	177.9(5)
O(3)-U(3)-O(9)	177.1(5)	O(5)-P(1)-O(6)	111.7(6)
O(5)-P(1)-O(12)	111.5(5)	O(6)-P(1)-O(12)	110.5(5)
O(5)-P(1)-O(18)	107.1(5)	O(6)-P(1)-O(18)	105.8(5)
O(12)-P(1)-O(18)	110.0(5)	O(1)-P(2)-O(13)	110.9(6)
O(1)-P(2)-O(2)	111.8(6)	O(13)-P(2)-O(2)	112.0(6)
O(1)-P(2)-O(14)	110.5(5)	O(13)-P(2)-O(14)	111.0(5)
O(2)-P(2)-O(14)	100.1(5)	O(11)-P(3)-O(19)	111.5(6)
O(11)-P(3)-O(21)	112.1(6)	O(19)-P(3)-O(21)	113.3(6)
O(11)-P(3)-O(16)	110.3(5)	O(19)-P(3)-O(16)	108.5(5)
O(21)-P(3)-O(16)	100.6(4)	O(22)-P(4)-O(17)	111.7(5)
O(22)-P(4)-O(15)	113.3(5)	O(17)-P(4)-O(15)	111.1(5)
O(22)-P(4)-O(8)	110.6(5)	O(17)-P(4)-O(8)	110.0(5)
O(15)-P(4)-O(8)	99.5(5)	O(18)-B(1)-O(16)	117.1(11)
O(18)-B(1)-O(8)#11	112.6(11)	O(16)-B(1)-O(8)	106.4(10)
O(18)-B(1)-O(14)	109.7(10)	O(16)-B(1)-O(14)	104.0(10)
O(8)#11-B(1)-O(14)	106.3(10)	B(1)#1-O(8)-P(4)	123.4(8)
B(1)-O(14)-P(2)	128.1(8)	B(1)-O(16)-P(3)	129.5(8)

Symmetry transformations used to generate equivalent atoms: #1 y+3/2, -x+3/2, z-1/4; #2 -y+2, -x+1, -z+3/2; #3 x+1/2, -y+1/2, -z+7/4; #4 -x+5/2, y+1/2, -z+5/4; #5 -y+1, -x+2, -z+3/2; #6 -y+2, -x+2, -z+3/2;

#7 y+1/2, -x+3/2, z-1/4; #8 x-1/2, -y+1/2, -z+7/4; #9 -y+3/2, x-1/2, z+1/4; #10 -y+1, -x+1, -z+3/2; #11 -y+3/2, x-3/2, z+1/4; #12 -x+5/2,y-1/2,-z+5/4; #13 y+1, x-1, -z+2.

Table S2. Bond Valence Sum (BVS) calculations for all U, P and B sites in CUPB1.

**U1**		**BVS**	**U2**		**BVS**	**U3**		**BVS**	**B1**		**BVS**
O20	1.758	1.7528882	O4	1.776	1.69	O3	1.768	1.7185862	O18	1.38	0.975969128
O10	1.768	1.7185862	O17	2.284	0.62	O9	1.781	1.6749952	O16	1.47	0.765238624
O13	2.284	0.6198605	O15	2.393	0.5	O19	2.317	0.5807248	O14	1.57	0.584009612
O12	2.291	0.6113444	O7	1.775	1.69	O1	2.274	0.6322325	O8	1.491	0.723015713
O22	2.307	0.5923158	O5	2.294	0.61	O14	2.683	0.2817322			
O21	2.402	0.4909257	O8	2.648	0.3	O6	2.301	0.5993811		B1-O	3.048233076
O16	2.648	0.3019094	O11	2.321	0.58	O2	2.384	0.5087038			
U1-O		6.0878301	U2-O		5.99	U3-O		5.9963558			

**P1**		**BVS**	**P2**		**BVS**	**P3**		**BVS**	**P4**		**BVS**
O5	1.505	1.353512	O1	1.498	1.3794	O21	1.51	1.3356692	O22	1.503	1.3608481
O18	1.551	1.1952775	O2	1.506	1.3499	O11	1.493	1.3981293	O8	1.604	1.0357597
O6	1.521	1.2962292	O13	1.504	1.3572	O16	1.58	1.1051709	O17	1.503	1.3608481
O12	1.519	1.3032548	O14	1.585	1.0903	O19	1.501	1.368224	O15	1.518	1.3067819
P1-O		5.1482736	P2-O		5.1767	P3-O		5.2071934	P4-O		5.0642378

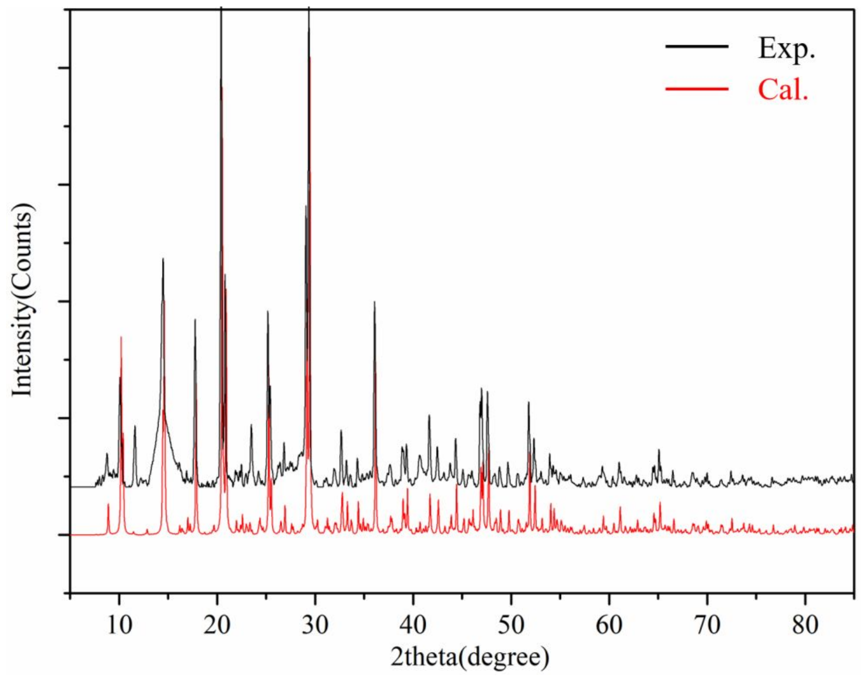

Figure S1. Experimental and calculated XRD patterns of **CUPB1**.

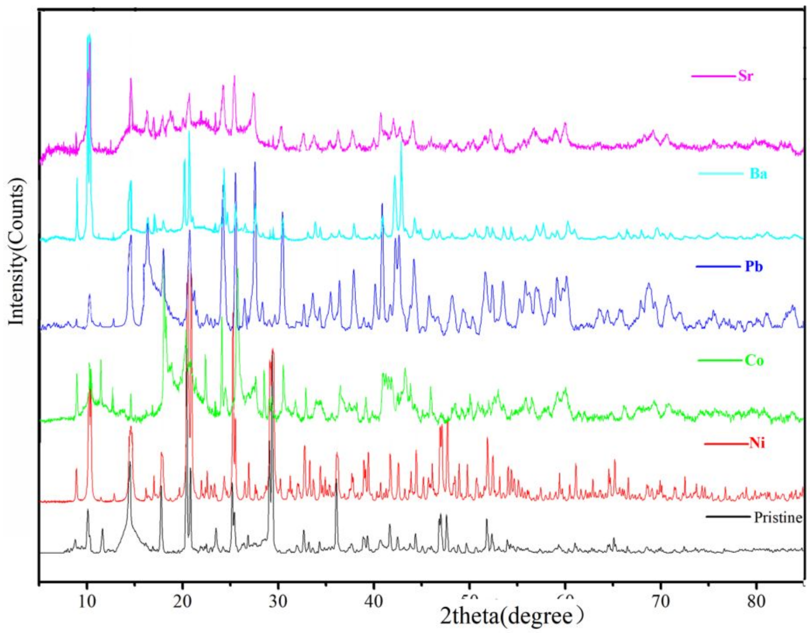

Figure S2. Pristine and Ion-exchanged XRD patterns for **CUPB1.**

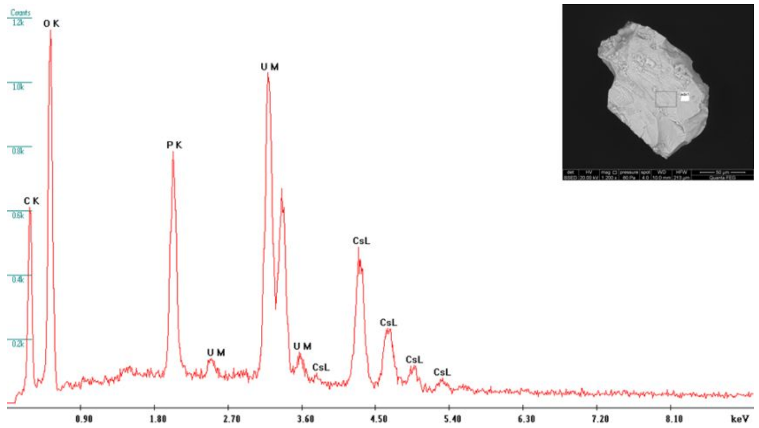

(a)

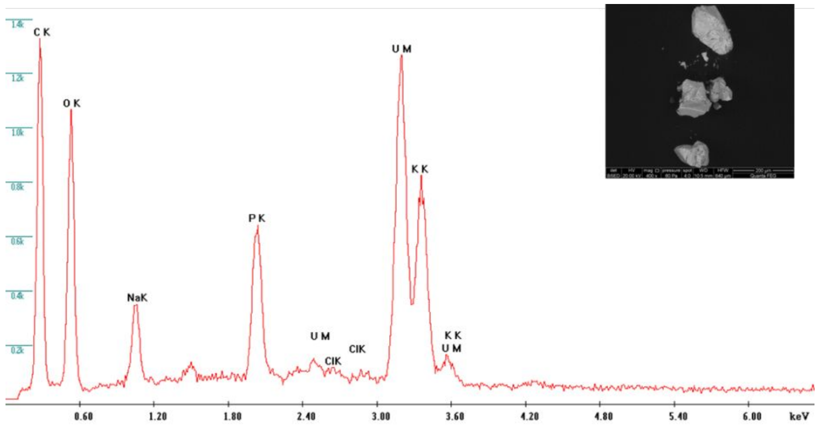
C K
O K
NaK
P K
U M
ClK
ClK
U M
K K
K K
U M
0.60
1.20
1.80
2.40
3.00
3.60
4.20
4.80
5.40
6.00
keV

(b)

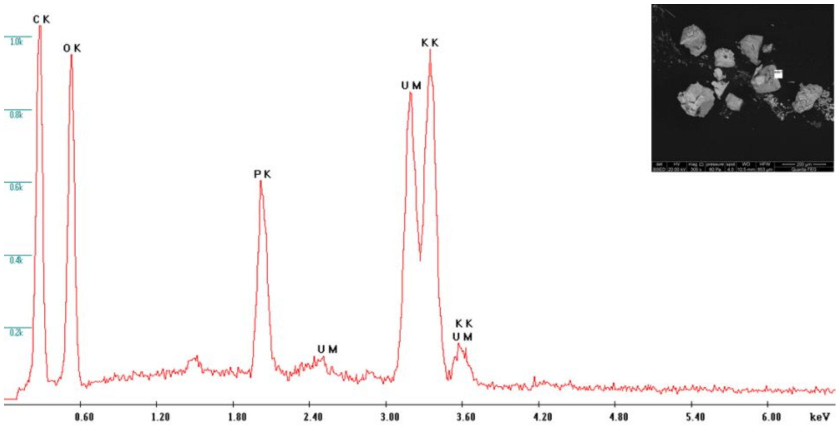
C K
O K
P K
U M
U M
K K
K K
U M
0.60
1.20
1.80
2.40
3.00
3.60
4.20
4.80
5.40
6.00
keV

(c)

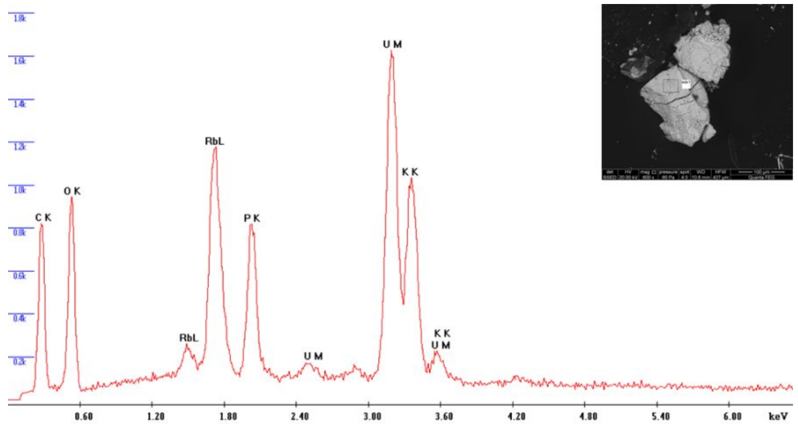
C K
O K
RbL
RbL
P K
U M
U M
K K
K K
U M
0.60
1.20
1.80
2.40
3.00
3.60
4.20
4.80
5.40
6.00
keV

(d)

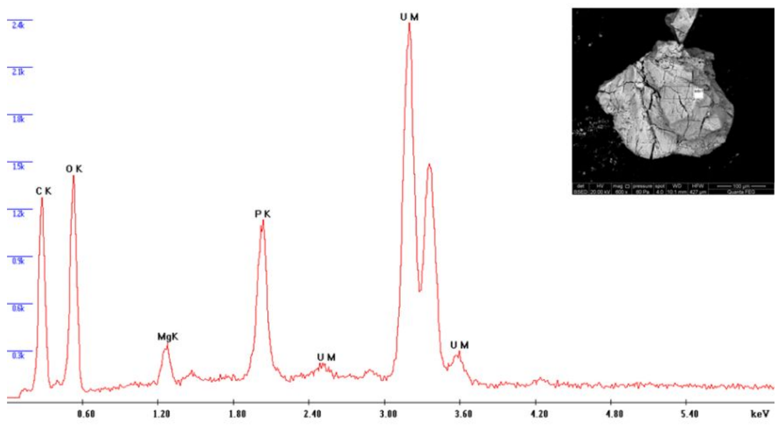
U M
O K
C K
P K
MgK
U M
U M
0.60
1.20
1.80
2.40
3.00
3.60
4.20
4.80
5.40
keV

(e)

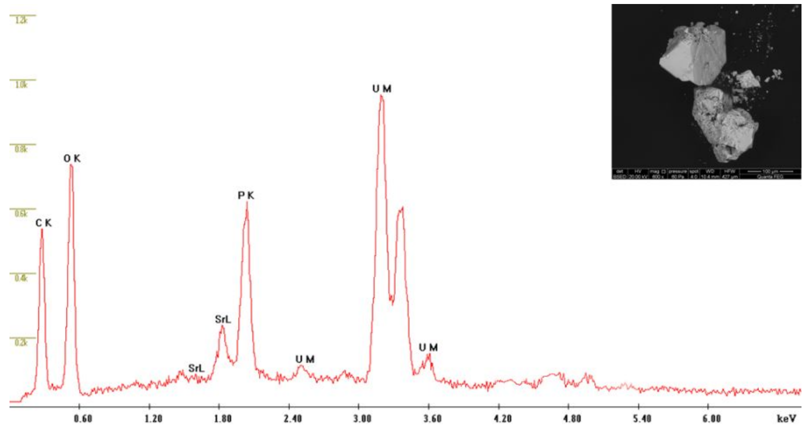
U M
O K
P K
C K
SrL
SrL
U M
U M
0.60
1.20
1.80
2.40
3.00
3.60
4.20
4.80
5.40
6.00
keV

(f)

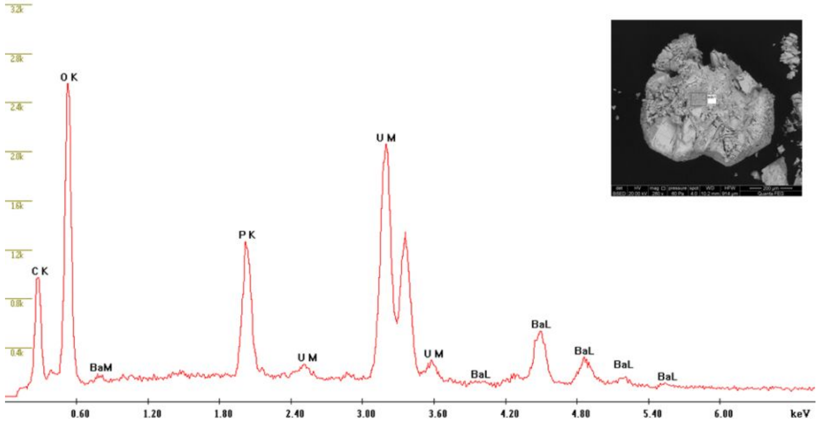
O K
U M
P K
C K
BaL
BaL
BaM
U M
U M
BaL
BaL
BaL
0.60
1.20
1.80
2.40
3.00
3.60
4.20
4.80
5.40
6.00
keV

(g)

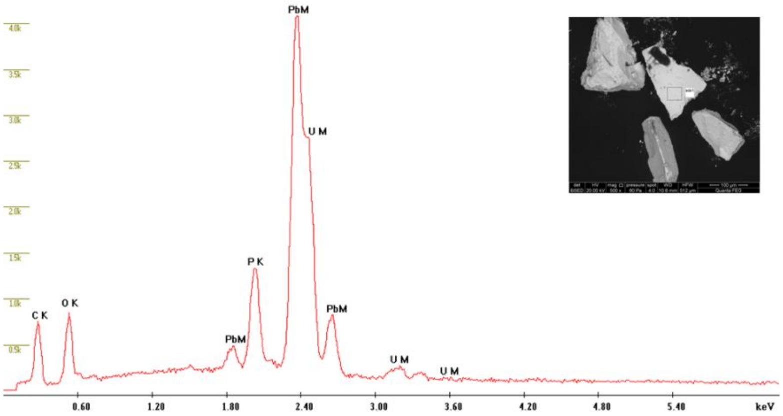

(h)

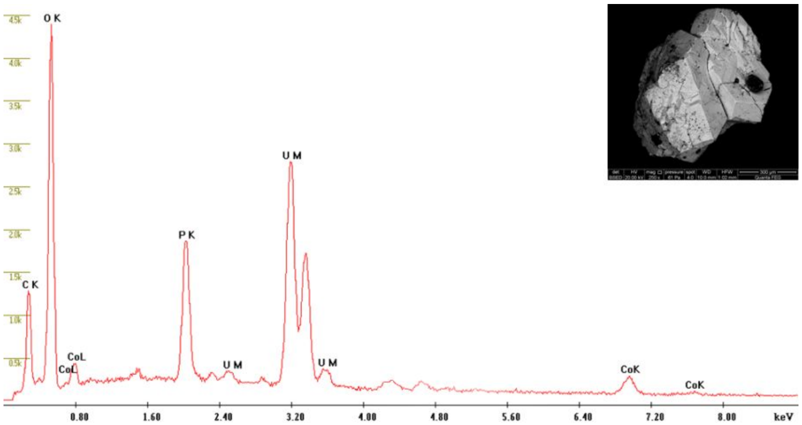

(i)

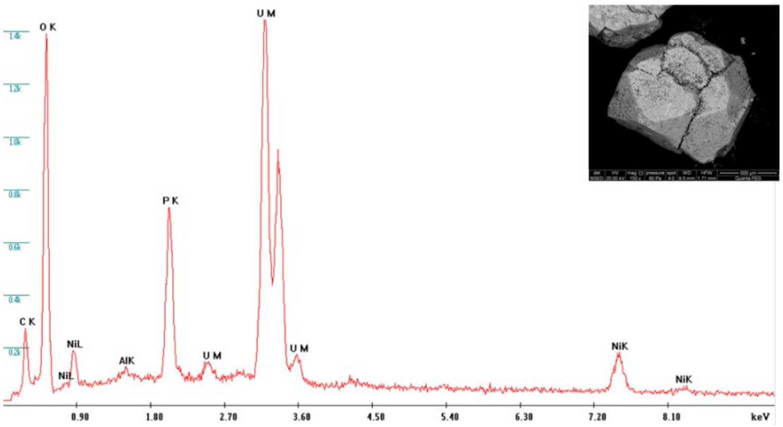

(j)

Figure S3. SEM and EDX measurements for pristine crystal **CUPB1** (a), and Na (b), K (c), Rb (d), Mg (e), Sr (f), Ba (g), Pb (h), Co (i), Ni (j)-exchanged samples.

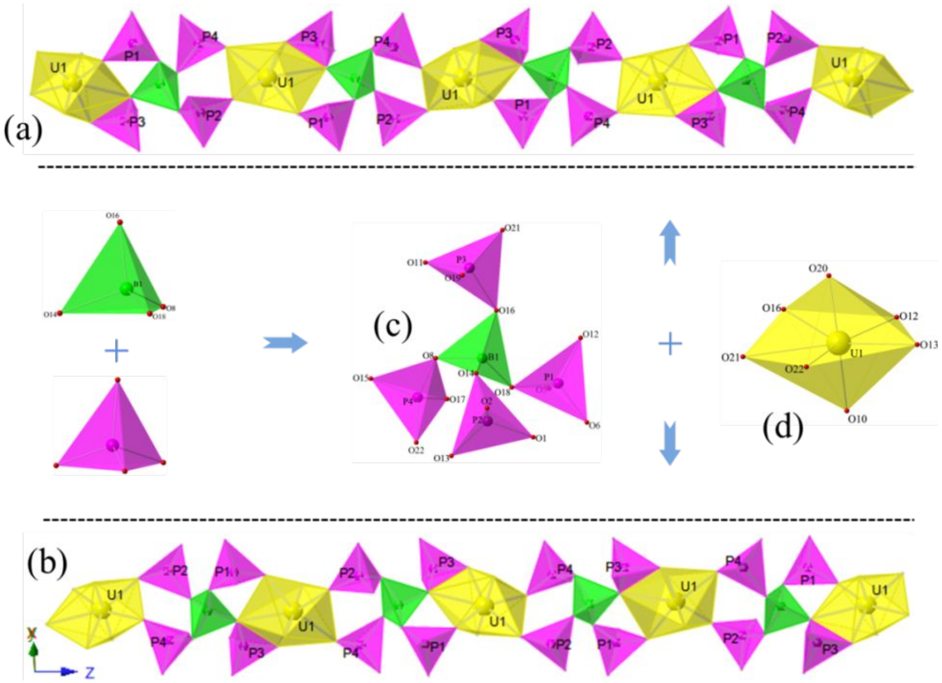

Figure. S4 Two chiral symmetrical uranyl borophosphate chains, (a) and (b); a FBB of $[B(PO_4)_4]$ (c); a $U(1)O_7$ pentagonal bypiramid. The UO_7 polyhedra, BO_4, PO_4 tetrahedra and O atoms are yellow, green, pink and red, respectively.

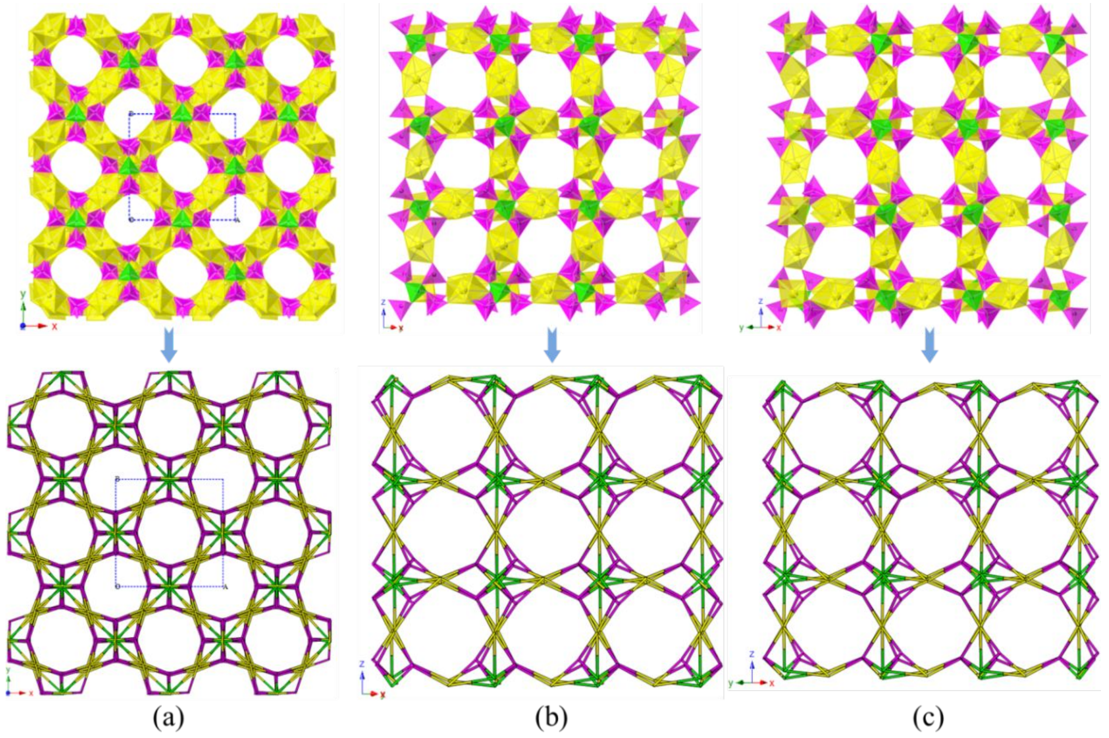

Figure. S5 View of the 3D framework of CUPB1 and its corresponding topology representations.

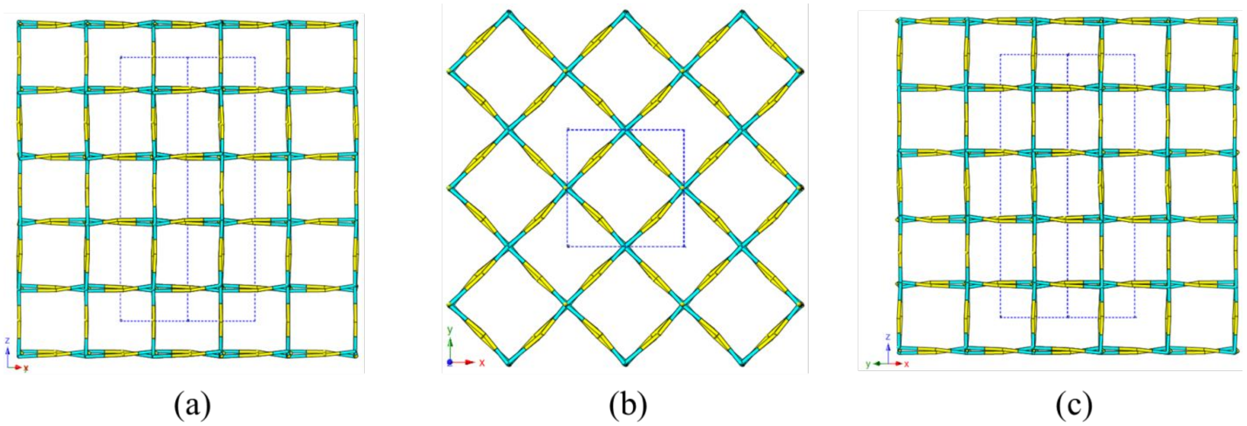

Figure S6. The simplified topology of the **CUPB1** network with a $X\mathrm{U}_3$ type structure.

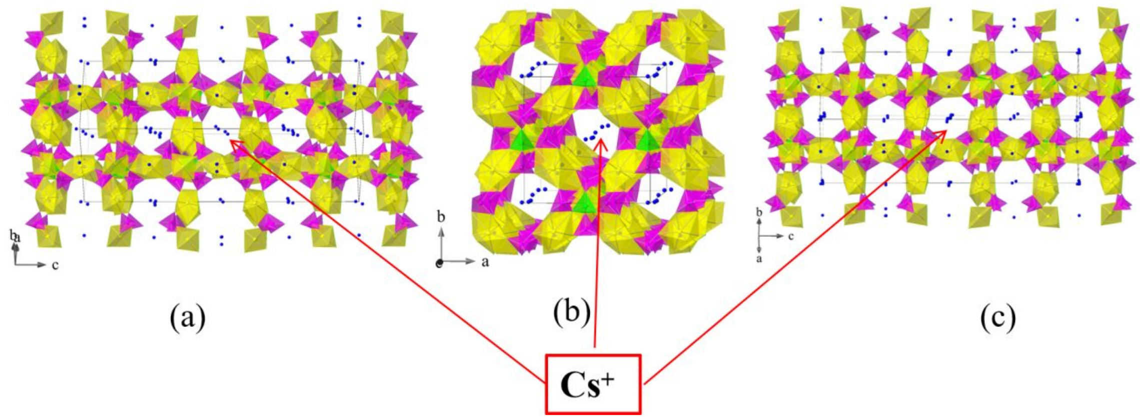

Figure S7. The locations of Cs+ in the intersecting open channel system along the (a) (1,-1,0); (b)(0,0,1) and (c) (1,1,0) directions.

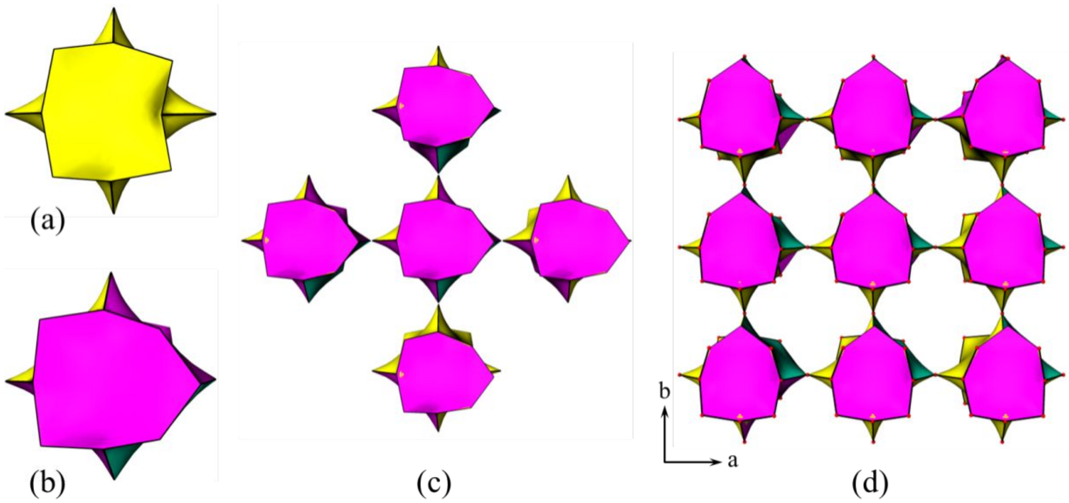

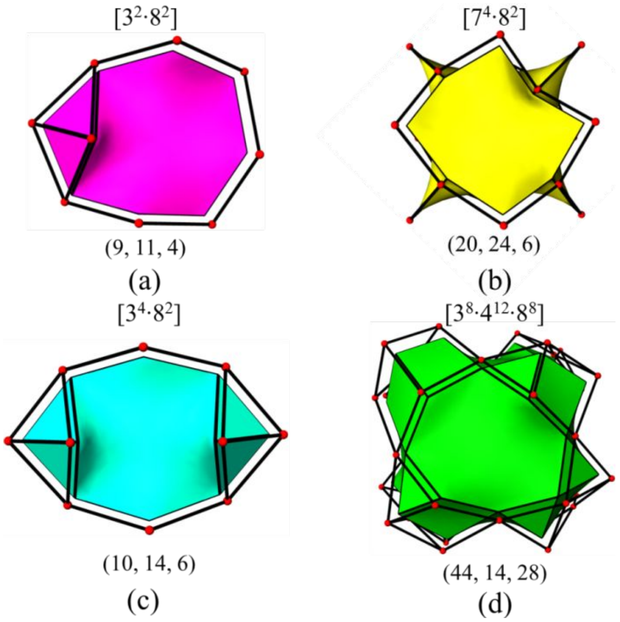

Figure S8. The constructions of the framework of CUPB1 using tilings, (a)-(d), and the details of all the basic tiles with the symbol of (V, E, F) (V = vertex, E = edge, and F = face).

Table S3. The data recorded for the concentration of A^{2+} (A = Sr, Ba, Pb, Co, Ni, UO_2) (C_A) and the relative amount of A-removed (R) at the different time in kinetic experiments from ICP-MS measurements.

Time (hour)	C_{Sr} (ppm) (RT)	~R(%) (RT)	C_{Sr} (ppm) (70°C)	~R(%) (70°C)
0	450(10)	0	450(10)	0
1.5	350(8)	22.2(4)	330(8)	26.7(3)
4	295(7)	34.4(3)	250(6)	44.4(4)
21	200(5)	55.5(2)	160(4)	64.4(2)
26	175(4)	61.1(2)	144(3)	68.0(1)
45	149(3)	66.8(2)	110(2)	75.6(2)
48	144(3)	68.0(1)	102(2)	77.3(1)
52	140(3)	68.9(1)	97(2)	78.4(2)
69	136(2)	69.8(2)	80(1)	82.2(2)
74	134(2)	70.2(2)	78(1)	82.7(2)

Time (hour)	C_{Ba} (ppm) (RT)	R(%) (RT)	C_{Ba} (ppm) (70°C)	R(%) (70°C)
0	703(13)	0	703(13)	0
1.5	626(12)	11.0(2)	610(12)	13.2(2)
4	585(10)	16.8(2)	566(11)	19.5(1)
21	436(10)	38.0(2)	405(9)	42.4(2)
26	398(8)	43.3(1)	366(8)	47.9(1)
45	289(7)	58.9(2)	252(6)	64.2(2)
48	268(7)	61.9(1)	230(6)	67.3(1)
52	249(6)	64.6(1)	208(5)	70.4(1)
69	207(5)	70.6(1)	146(3)	79.2(2)
74	201(5)	71.4(1)	136(3)	80.7(1)

Time (hour)	C_{Pb} (ppm) (RT)	R(%) (RT)	C_{Pb} (ppm) (70°C)	R(%) (70°C)
0	950(14)	0	950(14)	0
1.5	844(13)	11.1(5)	814(12)	14.3(4)
4	754(13)	20.6(5)	716(11)	24.6(5)
21	558(11)	41.2(2)	513(10)	46.0(2)
26	497(10)	47.7(4)	446(9)	53.1(4)
45	339(7)	64.3(2)	279(6)	70.6(2)
48	309(6)	67.5(1)	250(6)	73.7(1)
52	280(5)	70.5(2)	218(4)	77.1(3)
69	226(3)	76.2(3)	160(3)	83.2(3)
74	218(3)	77.1(2)	155(3)	83.7(2)

Time (hour)	C_{Co} (ppm) (RT)	*R*(%) (RT)	C_{Co} (ppm) (70°C)	*R*(%) (70°C)
0	300(6)	0	300(6)	0
1.5	255(5)	15.0(3)	236(4)	21.3(3)
4	231(5)	23.0(3)	205(3)	31.7(3)
21	172(3)	42.7(3)	158(2)	47.3(2)
26	161(3)	46.3(2)	147(2)	51.0(3)
45	132(2)	56.0(2)	111(2)	63.0(2)
48	126(2)	58.0(1)	105(2)	65.0(1)
52	120(2)	60.0(1)	98(1)	67.3(2)
69	110(2)	63.3(2)	82(1)	72.7(2)
74	107(2)	64.3(2)	78(1)	74.0(2)

Time (hour)	C_{Ni} (ppm) (RT)	*R*(%) (RT)	C_{Ni} (ppm) (70°C)	*R*(%) (70°C)
0	300(6)	0	300(6)	0
1.5	248(5)	17.3(4)	237(5)	21.0(3)
4	219(4)	27.0(3)	212(4)	29.3(4)
21	167(3)	44.3(3)	151(2)	49.7(2)
26	153(2)	49.0(3)	136(2)	54.7(4)
45	120(2)	60.0(2)	104(2)	65.3(3)
48	116(2)	61.3(2)	99(2)	67.0(2)
52	109(2)	63.7(1)	95(2)	68.3(2)
69	101(1)	66.3(2)	77(1)	74.3(2)
74	97(1)	67.7(2)	73(1)	75.7(2)

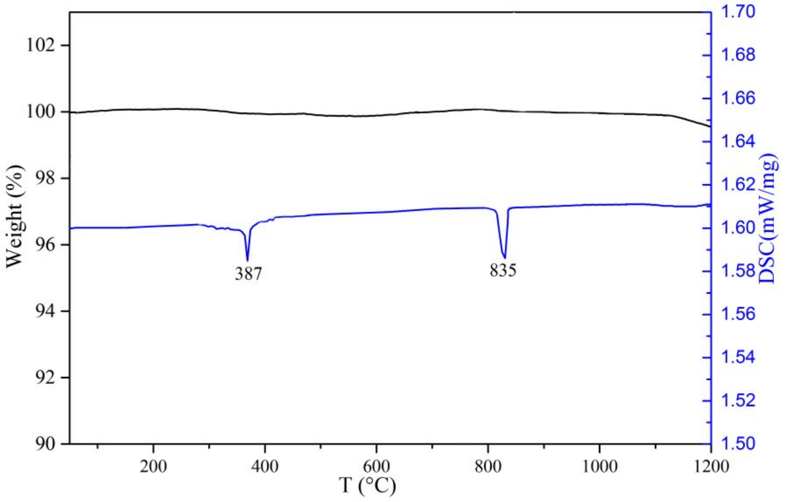

Figure S9. TG-DSC curves of **CUPB1**.

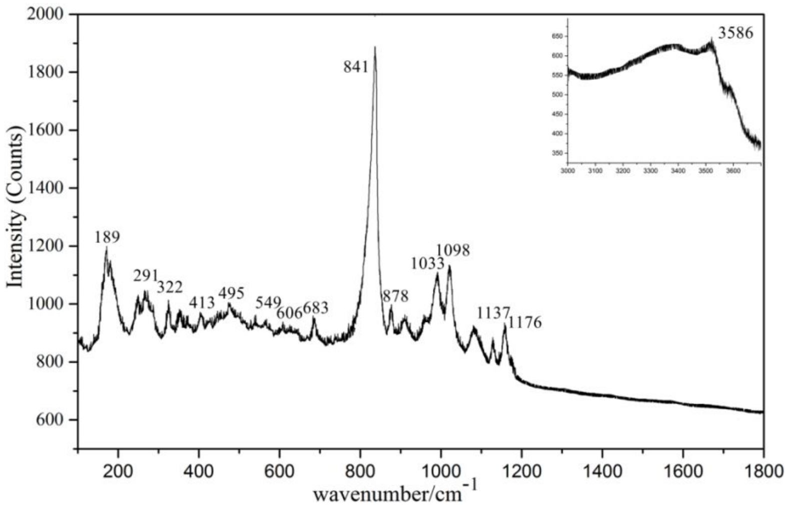

Figure S10. Raman shift of **CUPB1**.
